# Critical roles and clinical perspectives of RNA methylation in cancer

**DOI:** 10.1002/mco2.559

**Published:** 2024-05-07

**Authors:** Ganglei Li, Qinfan Yao, Peixi Liu, Hongfei Zhang, Yingjun Liu, Sichen Li, Yuan Shi, Zongze Li, Wei Zhu

**Affiliations:** ^1^ Department of Neurosurgery Huashan Hospital, Fudan University Shanghai China; ^2^ National Center for Neurological Disorders Shanghai China; ^3^ Shanghai Key Laboratory of Brain Function and Restoration and Neural Regeneration Shanghai China; ^4^ Neurosurgical Institute of Fudan University Shanghai China; ^5^ Shanghai Clinical Medical Center of Neurosurgery Shanghai China; ^6^ Kidney Disease Center The First Affiliated Hospital Zhejiang University School of Medicine Hangzhou Zhejiang China

**Keywords:** cancer immunity, cancer metabolism, clinical application, RNA metabolism, RNA modification

## Abstract

RNA modification, especially RNA methylation, is a critical posttranscriptional process influencing cellular functions and disease progression, accounting for over 60% of all RNA modifications. It plays a significant role in RNA metabolism, affecting RNA processing, stability, and translation, thereby modulating gene expression and cell functions essential for proliferation, survival, and metastasis. Increasing studies have revealed the disruption in RNA metabolism mediated by RNA methylation has been implicated in various aspects of cancer progression, particularly in metabolic reprogramming and immunity. This disruption of RNA methylation has profound implications for tumor growth, metastasis, and therapy response. Herein, we elucidate the fundamental characteristics of RNA methylation and their impact on RNA metabolism and gene expression. We highlight the intricate relationship between RNA methylation, cancer metabolic reprogramming, and immunity, using the well‐characterized phenomenon of cancer metabolic reprogramming as a framework to discuss RNA methylation's specific roles and mechanisms in cancer progression. Furthermore, we explore the potential of targeting RNA methylation regulators as a novel approach for cancer therapy. By underscoring the complex mechanisms by which RNA methylation contributes to cancer progression, this review provides a foundation for developing new prognostic markers and therapeutic strategies aimed at modulating RNA methylation in cancer treatment.

## Graphical abstract

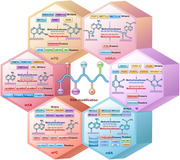

## 1 INTRODUCTION

Epigenetics is the study of the mechanisms through which behavior, environment, and phenotype alter gene expression.^1^, ^2^ Unlike genetic variations, epigenetic modifications are typically reversible. Epigenetic modification refers to heritable alterations in gene function that do not involve the alteration of the nucleotide sequence of a gene and that result in alterations in the genetic phenotype.^3^ Traditionally known epigenetic modifications include DNA methylation, histone modification, polymorphonuclear proteins, chromatin remodeling, and RNA interference.^4^, ^5^, ^6^ With advancements in chemistry, high‐throughput sequencing, and fluorescence quantification techniques, several types of RNA modifications have been discovered. In fact, pseudouridine (Ψ), a carbon–carbon‐linked ribonucleoside in ribonucleic acid, was discovered as early as 1960.^7^ Limited research has been conducted on RNA modification, which may be attributed to the fact that RNA, which lacks double chains in its structure, is less stable than DNA, and hence DNA, which carries genetic information, has garnered more attention from researchers. Additionally, the limited availability of suitable technology has also constrained research on RNA. At present, RNA modification, especially RNA methylation modification, is a prominent area of research.^8^, ^9^ To date, more than 100 distinct varieties of RNA modifications have been identified. All four RNA bases (A, C, G, and U) and ribose can be targeted for modification, and all known RNA species can be modified.^10^, ^11^, ^12^ RNA methylation accounts for 60% of RNA modifications. Mammalian RNA methylation modifications primarily include N^6^‐methyladenosine (m^6^A), N^1^‐methyladenosine (m^1^A), N6,2′‐O‐dimethyladenosine (m^6^Am), 7‐methylguanine (m^7^G), Ψ, and 5‐methylcytosine (m^5^C).^13^ Notably, DNA chemical modifications are named similarly, such as 5‐methylcytidine (5mC) in DNA and m^5^C in RNA. An emerging mechanism research has underscored the regulatory functions of RNA methylation on various RNA metabolism, including RNA splicing, export, stability, translation, and degradation.^14^, ^15^

Cancer is essentially a disease caused by mutations in oncogenes or tumor suppressor genes. Currently, it is one of the leading diseases contributing to human deaths worldwide.^16^, ^17^, ^18^ The basic signs of cancer are closely associated with the metabolism of cancer cells.^19^ Cancer metabolic reprogramming refers to the reprogramming of certain metabolic pathways in cancer cells. Metabolic reprogramming confers upon cancer cells a greater ability for survival and proliferation. As cancer progresses, cancer cells are more likely to undergo mutation, which can promote metabolic reprogramming, thereby establishing a robust positive feedback loop.^20^, ^21^ Cancer metabolism was first observed by Otto Warburg. Compared with normal tissue, cancer tissues consume considerable amounts of glucose to produce lactic acid in vitro, even in the presence of oxygen. This phenomenon is referred to as aerobic glycolysis or the Warburg effect.^22^, ^23^ The discovery of aerobic glycolysis in tumor cells has greatly contributed to advancements in the field of tumor metabolism. In addition to glycolysis, various metabolic processes, such as lipid metabolism, amino acid metabolism, mitochondrial metabolism, and iron metabolism, play notable roles in the development of tumors.^21^ The role of RNA methylation and cancer metabolism in cancer progression is apparent, and the relationship between RNA methylation and cancer progression has become a prominent area of research in recent years. Interestingly, researchers have found a synergistic relationship between RNA methylation and cancer metabolism.^24^, ^25^, ^26^ RNA methylation regulators have been found to activate or inhibit metabolic signaling pathways by modulating the expression of metabolism‐related genes, thereby playing a role in promoting or inhibiting cancer. RNA methylation has been observed in the abnormal metabolism of multiple types of cancer, which highlights the indispensability of the RNA methylation for cancer metabolism.^12^ Increasing evidence has suggested that the link of dysregulation of RNA methylation to cancer strongly supports the fruitful value of developing inhibitors targeting RNA methylation under the cancer condition.

Additionally, it is now becoming clear that cancer cells possess several distinct characteristics that endow them to proliferate uncontrollably, one of the essential hallmarks is immune escape.^27^, ^28^ By escaping from immune surveillance, cancer cells usually exhibit malignant abilities to escape from immune surveillance recognized and even destroy the immune balance of the organism.^29^ The impaired crosstalk between cancer cells and immune cells contributes to the continued cancer cell proliferation and distant tissue invasion in cancer progression.^30^, ^31^, ^32^ Understanding the mechanisms of immune evasion employed by cancer cells is crucial for developing effective strategies to enhance the immune response against cancer.^33^, ^34^, ^35^, ^36^ RNA modifications, such as m^6^A, m^5^C, m^7^G, and m^1^A, have been extensively revealed to participate in regulating tumor immunogenicity and the functions of diverse immune cells.^37^, ^38^ Several studies have shed light on the intricate networks of RNA methylation with immune cells, which disturbs the efficacy of immunotherapy mainly by influencing the programmed cell death protein 1 (PD‐1)/PD‐L1 signaling pathway in multiple cancers.^39^, ^40^, ^41^, ^42^ The exploration of RNA methylation on cancer immunity provides an excellent opportunity to exploit the RNA methylation‐driven clinical use in various malignancies.

In this review, we provide a comprehensive overview of several common RNA methylation, its underlying regulatory mechanisms on RNA metabolism, and the associated expression changes of genes involved in carcinogenesis. Then, we delve into recent advancements in understanding the roles and clinical applications of RNA methylation in cancer, using well‐studied cancer metabolism as an example. We highlight the challenges of identifying the predictive values of RNA methylation and developing targeted RNA modification‐based tools for cancer treatment concerning the context‐specific roles of RNA methylation in different types of cancer. Finally, we also briefly discuss the implications of RNA methylation with cancer immunity. We anticipate that our review will broaden the scope of research on RNA methylation in cancer and provide novel avenues for diagnosing, treating, and preventing various cancer types.

## 2 RNA METHYLATION MODIFICATIONS

**FIGURE 1:**
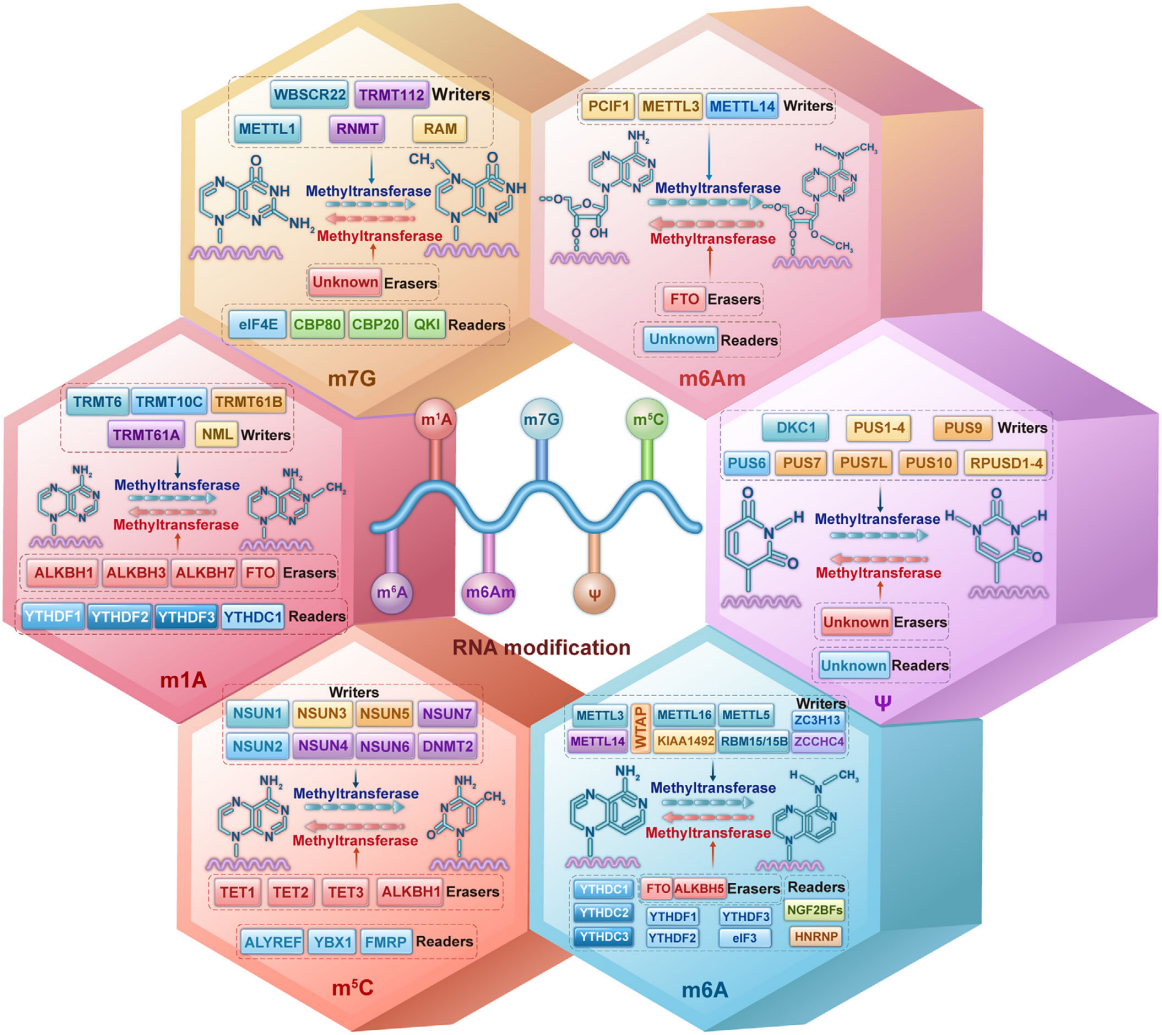
Overview of the mechanisms of m^6^A, m^1^A, m^6^Am, m^7^G, Ψ, and m^5^C regulation. RNA methylation is a dynamically reversible process that is modulated by methyltransferases (writers), demethylases (erasers), and recognition factors (readers). Under the regulation of these regulatory factors, RNA undergoes various RNA methylation modifications.

Chemical modifications of RNA affect RNA processing, localization, translation, and eventual decay through charge alterations, base pairing, secondary structure, and protein–RNA interactions. Alterations in the biological function of RNA regulate gene expression and ultimately play a role in growth, development, metabolism, and cancer, among others.^43^, ^44^ RNA methylation modification is the most prevalent form of RNA modification.^13^ In the subsequent sections, we describe several common RNA methylation modifications (Figure 1).

### 2.1 m^6^A

The most commonly observed RNA methylation modification is the m^6^A modification, accounting for 60% of all RNA methylation modifications. The m^6^A modification involves the methylation of the nitrogen‐6 position of adenosine and primarily detected within the RRACH sequence, with most sites located in close proximity to stop codons and within long internal exons. The m^6^A modification is present in almost all RNA and modulates the maturation, transcription, and functional metabolism of various classes of RNA, thereby regulating various cellular processes.^45^ For example, m^6^A affects protein encoding and synthesis by influencing both the translation and degradation of mRNA.^46^ The m^6^A modification of 18S rRNA and 28S rRNA is also necessary for global translation.^47^, ^48^ The m^6^A modification also facilitates the processing of microRNA (miRNA) precursors by recruiting the microprocessor complex protein DiGeorge syndrome critical region 8 (DGCR8) from the reading protein heterogeneous nuclear ribonucleoprotein A2/B1 (HNRNPA2B1).^15^ Furthermore, the m^6^A modification upregulates or downregulates the expression of both long noncoding RNAs (lncRNAs) and circular RNAs (circRNAs) through methylation modification, which has been observed to interfere with the progress of various diseases, including cancer. The m^6^A modification is a dynamically reversible process that is primarily accomplished through the collaborative efforts of methyltransferases (“writers”), demethylases (“erasers”), and effector proteins (“readers”). To date, several methyltransferases and effector proteins have been identified; however, research on demethylases has been relatively limited.

The m^6^A methyltransferases reported to date primarily include methyltransferase‐like 3 (METTL3), METTL14, METTL5, METTL16, RNA‐binding motif protein 15/15B, zinc‐containing finger CCCH‐type 13 (ZC3H13), zinc‐containing finger CCHC‐type 4, Wilms tumor 1‐associated protein (WTAP), and Vir‐like m^6^A‐related methyltransferase (also known as KIAA1429).^49^ METTL3 and METTL14 are the most widely observed m^6^A methyltransferases. METTL3 catalyzes the transfer of methyl to the N6‐adenosine residue of RNA. The primary function of the latter is to stabilize the methyltransferase complex and facilitate the recognition of catalytic substrates by METTL3.^50^ As for the demethylases, their primary role is to reversibly remove the m^6^A modification site on RNA, thereby mitigating the effect of methylation modification to varying degrees and ultimately maintaining the dynamic equilibrium of m^6^A modifications. Two demethylases, namely FTO alpha‐ketoglutarate‐dependent dioxygenase (FTO) and alkylation repair homolog protein 5 (ALKBH5), have been extensively studied. Methylated reading proteins primarily recognize and bind to m^6^A‐modified bases, thereby activating downstream regulatory pathways such as RNA degradation and miRNA processing pathways. Common reading proteins primarily include YT521‐B homology (YTH) domain proteins, eukaryotic initiation factor (eIF) 3, IGF2 mRNA binding protein (IGF2BP) family members, and heterogeneous ribonucleoprotein (HNRNP) protein family members. YTHDF1 and YTHDF3, which belong to the YTH family of proteins, interact with m^6^A‐modified RNA to initiate RNA translation. YTHDF2 primarily regulates the degradation of m^6^A‐modified RNA, whereas YTHDC1 promotes RNA splicing and export. YTHDC2 enhances target RNA translation and reduces target RNA abundance.^51^, ^52^, ^53^, ^54^, ^55^

### 2.2 m^1^A

The m^1^A modification refers to the methylation of the nitrogen atom located at position 1 of the adenine residue in the RNA molecule.^56^, ^57^ It was originally identified as a posttranscriptional modification that was highly prevalent in eukaryotic tRNA and rRNA.^58^, ^59^, ^60^ In archaea, bacteria, and eukaryotes, the m^1^A modification is often observed at positions 9, 14, 16, 22, 57, and 58 of the tRNA. Of these, m^1^A57 has been observed solely in archaea, m^1^A22 solely in bacterial tRNA, and m^1^A14 solely in cyt(tRNA)^Phe^ in mammals.^61^, ^62^, ^63^, ^64^ In human mitochondrial and cytoplasmic tRNAs, m^1^A is observed primarily at two sites, that is, positions 9 and 58.^65^, ^66^, ^67^ Regarding rRNA, m^1^A is primarily observed at position 645 of the 25S rRNA and position 1322 of the 28S rRNA.^68^, ^69^, ^70^ In addition, researchers have found that m^1^A plays various roles and exerts diverse effects on tRNA and rRNA. The m^1^A modification in tRNA is associated with the response to environmental stress, whereas in rRNA, it is primarily associated with the biogenesis of ribosomes and antibiotic resistance in bacteria.^71^, ^72^, ^73^ Recently, the m^1^A modification has also been found to be involved in the modification of mRNA. Studies have shown that the abundance of m^1^A‐modified mRNA is approximately 10‐fold lower than that of m^6^A‐modified mRNA.^74^, ^75^ The m^1^A modification has been observed in almost every fragment of mRNA, including the 5′ untranslated terminal region (UTR), the 3′ UTR, and the coding sequence (CDS).^76^, ^77^, ^78^ The 5′ UTR, the methyl donor for which is S‐adenosylmethionine, is most commonly found near the initiation codon.^74^, ^79^ Functionally, the m^1^A modification may potentially alter the structural stability of mRNA.^80^

Methyltransferases, demethylases, and RNA‐binding proteins are required to function collaboratively for the m^1^A modification to exert its effects. Researchers first discovered the tRNA m^1^A58 methyltransferase complex, a tetrameric enzyme consisting of two types of subunits (Gcd14p and Gcd10p, now designated Trm61 and Trm6), in *Saccharomyces cerevisiae*. Gcd14p plays a crucial role in the binding and catalytic function of AdoMet, whereas Gcd10p binds to tRNA.^81^, ^82^, ^83^ The TRMT6/TRMT61A complex is the homologous form of the tetrameric enzymes in eukaryotes. TRMT6/61A recognizes a GUUCRA tRNA‐like motif and promotes methylation of the m^1^A sites in mRNA. TRMT61B catalyzes the m^1^A modification of mt‐mRNA transcripts. TRMT10C methylates mt tRNA^Lys^ at positions m^1^A9 and ND5 mt‐mRNA 1374.^76^, ^77^, ^84^ Regarding rRNA, Rrp8 and Bmt2 catalyze m^1^A645 and m^1^A2142 modifications of 25S rRNA, respectively.^71^, ^85^ Nucleomethyl (NML) is a m^1^A‐modified nucleolar factor that catalyzes m1A modifications of 28S rRNA in human and mouse cells.^86^ In addition, demethylase and RNA methylated reading proteins function in a similar manner as m^6^A. To summarize, the m^1^A methyltransferases (writers) in eukaryotes primarily include TRMT6, TRMT61A, TRMT61B, TRMT10C, SDR5C1, and NML. Demethylases (erasers) primarily include alpha‐ketoglutarate‐dependent dioxygenase ABH1 (ALKBH1), ALKBH3, ALKBH7, and FTO. Methylated reading proteins (readers) primarily include YTHDF1‐3 and YTHDC1. Understanding the common sites of m^1^A modification and the types and functions of m^1^A‐modified proteins will enhance our understanding of this methylation modification and establish a basis for identifying suitable therapeutic targets.^57^, ^87^

### 2.3 m^6^Am

As a result of the methylation of 2‐O‐methyladenosine, m^6^Am is a modification of the first nucleotide proximal to the 7‐methylguanosine cap.^88^, ^89^ This modification was first discovered in viral mRNA and animal cells in the 1970s.^90^, ^91^ Although the formation of m^6^Am and m^6^A is similar, there are a few obvious differences between the two. First, the m^6^A modification sites are often situated in the 3′ UTR, whereas the m^6^Am modification is often detected in the 5′ m7G cap structure. Second, m^6^A exhibits various methyltransferases, including METTL3 and METTL14. Phosphorylated CTD interaction factor 1 (PCIF1) was traditionally considered the only m^6^Am methyltransferase. PCIF1 specifically recognizes the 5′ cap structure of mRNA and exhibits m^6^Am methyltransferase activity.^92^, ^93^, ^94^ However, studies in recent years have revealed that apart from PCIF1, METTL14 can also function as a m^6^Am “writer.” METTL4 catalyzes the methylation of the m^6^Am modification site in U2 small nuclear RNA (snRNA) and regulates pre‐mRNA splicing.^95^, ^96^ Third, while m^6^A can be demethylated by both ALKBH5 and FTO, m^6^Am is only preferentially and specifically demethylated by FTO.^97^, ^98^ At present, no regulatory protein for m^6^Am has been reported, and additional investigations are needed to delve into this unexplored area of research in the future. In general, m^6^Am is also a crucial RNA methylation modification in eukaryotes. m^6^Am governs the biosynthesis of snRNA, participates in the regulation of RNA splicing, and extensively affects mRNA cap‐dependent translation and stability, thereby affecting human diseases, including cancer.^98^, ^99^, ^100^, ^101^

### 2.4 m^7^G

The m^7^G modification refers to the addition of a methyl group at the N7 position of ribonucleoside, existing at internal positions in both tRNA and rRNA.^102^ Extensive research has revealed that levels of m^7^G modification are similar to those of m^1^A modification, that is, approximately 0.04% of all guanosine residues are modified.^103^ The m^7^G modification is predominantly observed in the 5′ cap region, or the internal region of mRNA, in eukaryotes. It is also found in tRNA, rRNA, and miRNA.^104^, ^105^, ^106^, ^107^, ^108^ In mammals, the most common and well‐studied m^7^G methyltransferase is METTTL1, which binds to WD repeat domain 4 (WDR4) and participates in the regulation of the m^7^G modification of various RNAs, including tRNA and mRNA. METTL1, located at 12q13,^26^ is widely expressed in many types of tissues. METTL1 is regulated by protein kinase B (AKT) and ribosomal S6 kinase and plays a notable role in the self‐renewal of embryonic stem cells and cancer cells.^109^, ^110^, ^111^ Located at 21q22.3, WDR4 is associated with Down's syndrome, microcephalic primordial dwarfism, and Galloway–Mowat syndrome with mental retardation.^112^, ^113^, ^114^ The action sites of the METTL1/WDR4 complex are primarily located at the internal site of the mRNA, the G46 site of the tRNA, and the G‐quadruplex structure of the miRNA.^108^, ^115^, ^116^ Knocking out WDR4 significantly reduces the expression of METTL1. In addition, the Williams–Beuren syndrome chromosomal region 22 (WBSCR22) and tRNA methyltransferase activator subunits 11−2 have been demonstrated to be responsible for the m^7^G modification of target rRNA.^116^, ^117^ The WBSCR22/TRMT112 complex primarily regulates the m^7^G modification of 18s rRNA to promote the maturation of 18s rRNA. The two methylase complexes are homologues of the Trm8p/Trm82p heterodimer complex and Bud23/Trm112 in yeast, respectively. In addition, RNA guanine‐7 methyltransferase (RNMT) and its cofactor, RNMT‐activated small protein (RAM), are also common m^7^G methyltransferases.^118^ This complex is primarily involved in the m^7^G modification at the 5′ cap of mRNA, thereby mediating the nuclear output of mRNA and translation. RAM, which consists of an N‐terminal RNMT activation domain and a C‐terminal RNA binding domain, increases the affinity of RNMT for RNA, thereby indirectly participating in the maintenance of mRNA levels and mRNA translation.^119^ To date, no definitive studies have reported m^7^G demethylases. As for “readers,” the m^7^G cap has been reported to be recognized by eIF4E and the cap‐binding complex consisting of CBP80 and CBP20, thereby affecting RNA maturation, nuclear output, and translation.^45^, ^120^ Zhao et al.^121^ elucidated that Quaking recognizes and binds to m^7^G‐modified mRNA.

### 2.5 Ψ

Ψ refers to a compound in which the C‐5 atom of the heterocycle is linked to the C‐1′ atom of pentose, unlike normal pyrimidine nucleosides in which the C‐1′ atom of pentose is linked to the N‐1 atom of the heterocycle to form a glycosidic bond.^122^ Ψ is the earliest identified modified nucleoside in RNA and exhibits the highest abundance. It is called the “fifth nucleoside” in RNA. To date, 13 writers of Ψ have been identified in humans, one of which is Dyskerin pseudouridine synthase 1 (DKC1) with an RNA‐dependent mechanism. DKC1 is the catalytic subunit of the H/ACA snoRNP complex and catalyzes the formation of Ψ in rRNA.^123^, ^124^ The other methylases in humans are primarily members of the PUS family, including PUS1‐4, PUS6, PUS7, PUS7L, PUS9, PUS10, and RPUSD1‐4.^125^, ^126^ To date, there have been no reports of Ψ erasers or readers. The lack of erasers may be owing to the inert C─C bond formed between a ribose and a base, leading to an irreversible pseudourification process. One of the key directions of future research will be to identify Ψ erasers and readers. In addition, given that Ψ is excreted directly through urine, it may serve as a promising biomarker for the diagnosis and treatment of cancer. It is worth mentioning that Ψ has been used to produce an effective COVID‐19 mRNA vaccine; therefore, it has notable research value.^127^

### 2.6 m^5^C

The m^5^C modification refers to the methylation of carbon located at position 5 in the cytosine residue of the RNA molecule.^128^ The m^5^C modification is widely observed in phenotypic transcriptomes, including RNA, such as mRNA, tRNA, rRNA, and enhancer RNA. m^5^C modification is most prevalent in tRNA and rRNA in eukaryotes.^129^ The m^5^C modification is relatively conserved in tRNA and rRNA and is primarily enriched in the 5′ UTR, CDS, and 3′ UTR in mRNA.^130^, ^131^ m^5^C methyltransferases primarily include DNA methyltransferase 2 (DNMT2) and members of the NOP2/SUN (NSUN) RNA methyltransferase family. The NSUN family has been most extensively studied for NSUN2, which modulates the m^5^C modification in diverse RNA transcripts, including tRNA, mRNA, rRNA, mitochondrial tRNA (mt‐tRNA), and vault‐derived small RNA.^13^ For instance, NSUN1 primarily regulates the m^5^C modification at position 4447 in human 28S rRNA and position 2870 in yeast 25S rRNA.^132^ NSUN3 initiates the biogenesis of m^5^C in mt‐tRNA (Met) in humans.^133^ Furthermore, NSUN4 and NSUN5 are associated with the m^5^C modification at position 911 in human 12S RNA and position 3782 in human 28S RNA, respectively.^134^, ^135^ NSUN6 primarily facilitates the m^5^C modification of tRNA (Cys) and tRNA (Thr) at cytosine C72.^136^, ^137^ DNMT2 (also known as TRMDT1) not only catalyzes tRNA methylation but also mRNA methylation.^138^ Erasers that have been identified thus far include the 10−11 translocation (Tet) family (Tet 1−3) and ALKBH1. The members of the Tet family successively oxidize 5mC to 5‐hydroxymethyl cytidine, 5‐formyl cytidine (5frC), and then to 5‐carboxy cytidine in RNA.^139^, ^140^ The m^5^C modification may undergo additional oxidation by ALKBH1/ABH1, resulting in the production of 5frC at the same position.^141^ The methylated reading proteins of m^5^C primarily include Aly/REF derivation factor (ALYREF, also known as THOC4) and Y‐box binding protein 1.^45^ More recently, Lan et al. elucidated a novel m^5^C interpreter, namely fragile x messenger ribonucleoprotein 1.^142^ This interpreter is recruited by DNMT2 to sites of DNA damage and facilitates the demethylation of Tet 1‐mediated m^5^C‐modified RNA in DNA:RNA hybrids.

## 3 RNA METHYLATION AND RNA METABOLISM

### 3.1 Regulation of RNA methylation on RNA metabolism

It has been extensively acknowledged that RNA methylation influences almost every step of RNA metabolism, from splicing and export in the nucleus, to translation as well as degradation in the cytoplasm.^143^, ^144^, ^145^

In terms of RNA splicing, previous studies have highlighted the role of m^6^A as a splicing regulator, facilitating the production of mature mRNAs by removing introns from precursor mRNA.^146^ Additionally, mRNAs generated through alternative splicing have been observed to possess more RNA methylation sites.^147^ FTO has been found to inversely control m^6^A levels around splice sites of Runt‐related transcription factor 1 (RUNX1) in an m^6^A demethylation manner, thereby restoring adipogenesis.^148^ Furthermore, HNRNPC has been shown to bind to the altered local structure in mRNA and lncRNA caused by m^6^A, thereby influencing alternative splicing and the abundance of target RNAs.^149^, ^150^ Research has also shown that m^1^A is observed in nascent polycistronic mitochondrial RNA and involves the processing of mitochondrial polycistronic RNAs.^57^, ^151^ Additionally, m^1^A induces the proper folding of tRNA, contributing to early tRNA maturation events.^66^, ^82^ Regarding RNA export, the protein ALKBH5 plays a significant role in mRNA export and the assembly of mRNA processing factors by colocalizing with nuclear speckles.^152^, ^153^ In addition, recent studies have reported that ALYREF specifically recognizes m^5^C modifications catalyzed by NSUN2, enhancing mRNA export.^130^, ^154^ In the case of RNA translation, YTHDF1 typically binds to m^6^A sites around stop codons in mRNA, recruiting translation initiation factors such as eukaryotic translation initiation factor 3 (eIF3), eukaryotic translation initiation factor 4E (eIF4E), eukaryotic translation initiation factor 4G (eIF4G), and the 40S ribosomal subunit to increase the translation efficiency of the target RNAs.^155^ In some cases, cap‐independent translation has also been observed near the 5′ UTR of RNA.^156^ Emerging studies suggest that mRNAs modified by m^6^A in their 5′ UTR can directly initiate translation by interacting with eIF3 and recruiting the 43S complex.^157^ Moreover, m^7^G methyltransferase METTL1/WDR4 complex also exerts essential effects on the process of mRNA translation, particularly for regulating cell cycle genes and brain abnormality‐related genes.^110^ It has also been indicated that m^1^A participates in the translation initiation and elongation process of tRNA, mRNA and rRNA.^62^, ^158^ As a m^1^A demethylase, ALKBH1 reversibly attenuates translation initiation and usage of tRNAs in protein synthesis by catalyzing the demethylation of the target tRNAs.^158^ In course of RNA degradation, YTHDF2 reduces the stability of target transcripts by recognizing m^6^A modification and eventually transporting RNA to the processing body (P‐body).^52^, ^147^, ^159^ Furthermore, m^1^A RNA‐binding protein HRSP12 has been identified as an adaptor that facilitates the connection between YTHDF2 and RNase P/MRP (endoribonucleases), thereby involving in the acceleration of mRNA degradation.^14^, ^160^, ^161^ It has also been indicated that METTL1 are responsible for the base triple interaction of tRNA variable loop, which influences the stability of tRNA.^162^, ^163^

### 3.2 Association of RNA methylation with abnormal RNA metabolism in cancer

Emerging evidence demonstrated that aberrant RNA methylation exerts profound influence on gene expression by modulating diverse processes in RNA metabolism, which provides abilities to developmental or environmental changes.^164^, ^165^, ^166^, ^167^ Accumulating evidence has revealed that dysregulation of RNA methylation affects numerous types of RNA processing and subsequently alters the expression of cancer‐associated genes, playing a pivotal role in the molecular events underlying carcinogenesis.^168^, ^169^, ^170^, ^171^

Numerous studies have established that multiple m^6^A regulators disturb cancer‐associated gene expression by influencing RNA splicing, translation, and stability, thereby performing oncogenic activities in cancer progression.^172^ Previous studies have reported that METTL3 methylates pri‐miR221/222, leading to enhanced recognition and combination by microprocessor protein DGCR8 of modified pri‐miR221/222. This process promotes the processing and maturation of pri‐miR221/222 in bladder cancer and highlights the oncogenic role of METTL3. Specifically, matured miR221/222 mediated by METTL3 inhibits the expression of phosphatase and tensin homolog (PTEN), contributing to the development and progression of bladder cancer.^173^ Recent evidence has shed light on the role of YTHDF2 in accelerating the mRNA degradation of phospholysine phosphohistidine inorganic pyrophosphate phosphatase (LHPP) and the prostate‐specific homeobox gene NKX3−1 and the consequent activation of Akt This acceleration occurs via METTL3‐mediated m^6^A‐dependent mechanisms and significantly enhances tumor growth and metastasis in prostate cancer (PCa). YTHDF2 has also been shown to induce METTL3‐mediated suppressor of cytokine signaling 2 (SOCS2) mRNA degradation in hepatocellular carcinoma (HCC), therefore driving HCC cell proliferation, migration, and colony formation.^174^ While ALKBH5 is indicated to perform vital inhibitory effects in HCC tumorigenesis. Through mediating demethylation on LY6/PLAUR Domain Containing 1 (LYPD1) mRNA to enhance its degradation, ALKBH5 attenuates the proliferation and invasion capabilities of HCC cells.^175^ As the most common and fatal types of hematopoietic malignancies, acute myeloid leukemia (AML) is recently reported to aberrantly upregulate FTO expression, especially in AML cases with t(11q23)/MLL‐rearranged chromosomal abnormalities. Elevated FTO level in AML cells suppresses the m^6^A abundance and stability of ankyrin repeat and SOCS box‐containing 2 (ASB2) and retinoic acid receptor alpha (RARA) mRNA. This suppression leads to cell transformation and leukemogenesis. It has also been reported that METTL14 increases stability and translational efficiency of MYB and MYC mRNAs in an RNA methylation‐dependent manner, thereby contributing to self‐renewal and proliferation of AML cells during malignant hematopoiesis.^176^

Growing evidence also suggests that abnormal m^5^C modification affects the expression of downstream cancer‐associated genes by altering related RNA metabolism processes. In cervical cancer (CC), NSUN2 and YBX1 were also observed to mediate m^5^C modification and strengthen the mRNA stability of LRRC8A, a core component of the volume‐regulated anion channel. Then, upregulated LRRC8A further activated PI3K/AKT signaling to exert its tumor‐promoting role in CC.^177^ In bladder cancer, m^5^C reader YBX1 recognized NSUN2‐induced m5C modification in 3′ UTR of hepatoma‐derived growth factor (HDGF) mRNA and further maintained its stability, therefore driving the oncogenic molecular mechanism of HDGF in bladder cancer.^178^ Research has also revealed the involvement of m^5^C in regulating RNA stability and ferroptosis during cancer progression. In endometrial cancer (EC), NSUN2 enhances the m^5^C modification of SLC7A11 mRNA, increasing its stability and expression levels. This leads to reduced susceptibility to ferroptosis and enhanced proliferation of EC cells.^179^

In addition, dysregulated m^7^G tRNA modifications have been implicated in aberrant mRNA translation and tumorigenesis in multiple cancers.^107^, ^162^, ^180^, ^181^, ^182^ Dysregulation of mRNA translation is a prominent feature of cancer, leading to the excessive synthesis of oncogenic proteins involved in various oncogenic processes.^183^, ^184^, ^185^ Cancer cells often exhibit altered mechanisms of translational control, enabling increased production of proteins that promote tumor growth, survival, angiogenesis, and metastasis.^186^, ^187^, ^188^, ^189^ Recent studies have highlighted the role of the METTL1/WDR4 complex in elevated mRNA translation and enhanced tRNA stability, which contributes to the development of several cancers. Notably, the upregulated expression of the METTL1/WDR4 complex has been found to drive translational regulation of specific oncogenic mRNAs in HCC through m^7^G tRNA modification‐dependent mechanisms, thereby fueling hepatocarcinogenesis. This dysregulation is accompanied by increased HCC cell growth, migration, and invasion.^190^, ^191^ In AML, the METTL1/WDR4 complex has been shown to positively control global translation efficiency, underscoring its role in leukemogenesis.^192^ Additionally, in the progression of head and neck squamous cell carcinoma (HNSCC), the upregulation of the METTL1/WDR4 complex enhances the translation of oncogenic transcripts associated with the PI3K/AKT/mTOR signaling pathway, leading to alterations in the immune landscape and subsequent malignant development of HNSCC.^193^

Recently, m^1^A methylation has also emerged as a novel layer of epigenetic regulation implicated in human carcinogenesis.^194^, ^195^ One example is the interaction between ribosomal RNA methyltransferase NSUN4 and mitochondrial transcription termination factor 4 (MTERF4) to form a stable complex. This complex is essential for the generation of functional ribosomes and translation in mammalian mitochondria.^196^ Moreover, tRNA methyltransferase TRM6/61 plays a role in stabilizing and accumulating charged initiator methionyl‐tRNA (tRNAi^Met^) by methylating its adenosine 58, therefore contributing to the increased translation of mRNA that are frequently deregulated in gliomas.^197^ Additionally, it has been confirmed that protein kinase C α (PKCα) is confirmed to restrain TRM61 activity and TRM61‐induced cancer‐promoting effects on anchorage‐independent growth and sphere‐forming ability of glioma C6 cells.^198^ Moreover, the knockdown of ALKBH3 in HEK293T cells has been observed to reduce the expression of epidermal growth factor receptor 2 (ErbB2) and AKT1 substrate 1 (AKT1S1), a potential role for ALKBH3 in the progression or regulation of gastrointestinal cancer.^199^ It has also demonstrated that ALKBH3 exerts the oncogenic role in the survival, angiogenesis, and invasion of urothelial carcinoma cells. Upregulated ALKBH3 has been found to significantly elevate the expression of downstream molecules, including NAD(P)H oxidase‐2 (NOX‐2) and TNF‐like weak inducer of apoptosis (Tweak), in urothelial carcinoma UMUC2 and UMUC3 cells, thereby inducing the accelerated cell cycle and angiogenesis in the development of urothelial carcinoma.^200^

These findings consistently emphasize the intricate associations between RNA methylation, RNA metabolism, and cancer development in different cellular contexts. Disruption of these processes contribute to the initiation and advancement of cancer. Gaining a deeper understanding of the mechanisms that underlie these connections could offer valuable insights into the development of innovative therapeutic approaches for cancer treatment.^201^

## 4 RNA METHYLATION AND CANCER METABOLISM

This section utilizes the extensively studied phenomenon of cancer metabolic reprogramming as a focal point to dissect the specific roles and mechanisms through which RNA methylation influences cancer biology. This exploration not only sheds light on the direct impact of RNA methylation on the metabolic pathways pivotal for cancer cell survival and proliferation but also emphasizes the translational potential for clinical applications of these findings. By understanding the intricacies of how RNA methylation regulates key metabolic processes in cancer, we pave the way for the development of RNA methylation‐targeted prognostic markers and therapeutic tools. The focus on tumor metabolism driven by RNA methylation underscores the potential of targeting RNA methylation as an innovative strategy in the clinical setting to improve cancer management and patient outcomes.

### 4.1 Cancer metabolism

**FIGURE 2:**
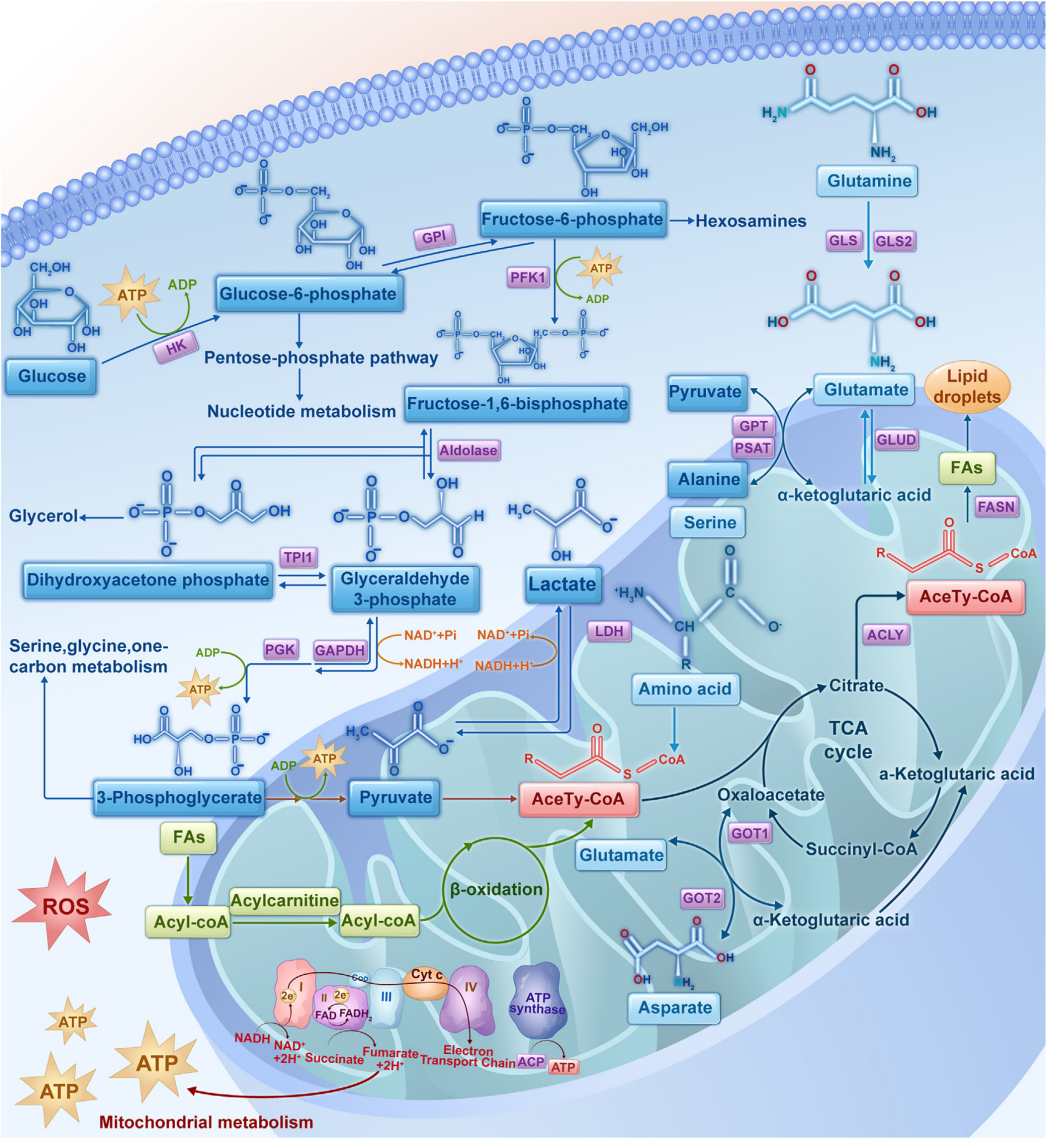
Metabolic reprogramming plays an indispensable role in tumorigenesis. Cancer cells often exhibit fiendishly complicated metabolic patterns and initiate the most suitable metabolic pathways to address the requirements of cell proliferation and survival in a hostile microenvironment. In contrast to normal cells, cancer cells predominantly rely on the enhanced glycolytic pathway to efficiently generate ATP at a rapid rate to meet the high energy demands associated with cancer growth. Cancer cells also exhibit increased uptake and metabolism of many other nutrients, including glutamine and FAs, thereby facilitating their proliferation and invasion. Additionally, cancer cells exhibit altered mitochondrial metabolism to meet the changeable need of energy generation and biosynthetic processes. FA, fatty acid; FASN, FA synthase; HK, hexokinase; GPI, glucose‐6‐phosphate isomerase; PFK1, phosphofructokinase 1; GPT, glutamic pyruvate transaminase; PSAT, phosphoserine aminotransferase; GLS, glutaminase; GLUD, glutamate dehydrogenase; TPO1, transporter protein 1; PGK, phosphoglycerate kinase; LDH, lactate dehydrogenase; GOT1, glutamic‐oxaloacetic transaminase 1; ACLY, ATP citrate lyase.

Energy metabolism is essential for all living organisms to sustain basal biological processes.^202^ In a broad sense, energy metabolism primarily encompasses the metabolism of carbohydrates, proteins, lipids, nucleic acids, and other substances. The interplay and influence of diverse metabolic substances, which undergo a series of biochemical reactions, involve various enzymes and substrates, thereby establishing an intricately interconnected metabolic network that drives various vital physiological processes throughout an organism's lifespan.^203^, ^204^ Unlike normal cells, cancer cells exhibit increased nutrient requirements, such as those for glucose, glutamine (Gln), and fatty acids (FAs), to generate ATP and meet high energy demands (Figure 2).^205^, ^206^ The concept of the “Warburg effect” was first proposed by Nobel laureate Otto Warburg in the 1920s and has since contributed to notable advancements in research on cancer metabolism.^207^, ^208^ Metabolic reprogramming exhibited by cancer cells has been recognized as one of the fundamental characteristics of cancer.^19^, ^20^ Interestingly, a metabolic trait observed in nearly all cancer cells is their ability to acquire nutrients from a nutrient‐deficient environment and utilize the nutrients to sustain their hyperproliferative capacity and biomass synthesis.^209^, ^210^ Cancer cells typically exhibit unique metabolic adaptations that switch different metabolic patterns to maximize the generation of energy and biomass. These adaptations involve the upregulation of glycolysis, glutaminolysis, lipogenesis, amino acid metabolism, mitochondrial metabolism, and nucleotide synthesis.^211^, ^212^, ^213^, ^214^ Studies have demonstrated that these biosynthetic processes involve the regulation of signaling pathways associated with cell growth. These signaling pathways (commonly PI3K/AKT/mTOR signaling) are activated as a result of tumorigenic mutations in the cancer cells.^209^ Metabolic reprogramming is accompanied by cancer initiation and development, with the enhancement of diverse neoplastic processes, including cell proliferation, survival, invasion, metastasis, and resistance to treatment. The progression of cancer is characterized by the acquisition of additional oncogenic mutations, which further enhance metabolic reprogramming and subsequently accelerate cancer growth.^215^ Notably, metabolic reprogramming is influenced by genetic alterations, the surrounding microenvironment, and their interactions.^216^ To address depleted supplies of precursors for macromolecule biosynthesis and cell survival, a few cancer‐related genes acquire specific mutations to drive metabolic reprogramming.^217^ Mutations in oncogenes, suppressor genes, and certain metabolic genes, such as MYC, RAS, p53, PTEN, EGFR, HIF‐1, isocitrate dehydrogenase (IDH) 1/2, and fumarate hydratase (FH), actuate diverse metabolic phenotypes and subsequently aberrantly change the tumor microenvironment (TME).^209^, ^218^, ^219^, ^220^ While nutrient availability and complex noncancer components in the TME influence the metabolic state of cancer cells and consequently lead to epigenetic alterations in these cells,^221^, ^222^, ^223^, ^224^, ^225^ the interactions between oncogenic mutations and the TME jointly endow cancer cells with advantageous metabolic traits.^226^, ^227^ Several studies have shown that the uptake of both glucose and Gln is notably higher than that of other nutrients in cancer cell. The increased uptake of glucose and Gln in cancer underscores the importance of energy and biosynthesis in cancer.^228^ Glucose and Gln metabolism are interconnected in various ways.^229^ Given the different tissue origins, oncogenic mutations, and nutrient availability in the TME, distinct cancer types and even intratumorally phenotypic states exhibit metabolic heterogeneity and distinct metabolic patterns.^230^, ^231^, ^232^, ^233^, ^234^ Therefore, we focus on the major metabolic processes associated with glucose, amino acids, lipids, and the mitochondria in cancer cells and summarize the mechanisms underlying classical and common metabolic pathways, taking into account the influence of both genetic and microenvironmental factors.

#### 4.1.1 Glucose metabolism

Glucose, the preferred energy and carbon source for cells, primarily undergoes glycolysis and enters the pentose phosphate pathway (PPP) in the cytoplasm or the tricarboxylic acid (TCA) cycle and oxidative phosphorylation (OXPHOS) in the mitochondria.^235^, ^236^, ^237^ The major portion of ATP required for cellular processes (approximately 70−80%) is produced via glucose metabolism through the OXPHOS pathway in the mitochondria.^238^, ^239^, ^240^ Glucose plays a central role in biosynthetic processes by serving as carbon skeletons (accounting for over 90%) and a source of reducing power.^213^ Glycolysis is the virtually ubiquitous metabolic pathway for glucose once it is transported into cells via glucose transporters (GLUTs).^241^ The OXPHOS pathway is another major pathway for glucose, where ATP is produced across the mitochondrial inner membrane with the consumption of oxygen.^242^ The dysregulation of glucose metabolism is a key characteristic of metabolic reprogramming during tumorigenesis. Aerobic glycolysis, which is widely recognized as the predominant metabolic pathway in cancer cells, has been extensively investigated to determine the association between metabolism and tumorigenesis, thereby providing insights into cancer metabolic reprogramming.^243^ During the early twentieth century, Otto Warburg demonstrated that in a large proportion of cancers, cancer cells exhibit increased glucose uptake and convert a considerable proportion of glucose into lactate, regardless of the availability of oxygen, in comparison with normal cells.^207^ Although decades of research have been devoted to determining the causes and mechanisms of upregulated aerobic glycolysis, the precise mechanism remains incompletely understood.^244^ Several widely recognized potential reasons for the upregulation of aerobic glycolysis in cancer cells include mitochondrial dysfunction, high‐efficiency ATP generation, the upregulation of oncogenes and glycolytic enzymes, and hypoxia.^245^ Warburg has postulated that the occurrence of aerobic glycolysis can be attributed, at least in part, to mitochondrial dysfunction leading to compromised aerobic respiration.^208^, ^246^ Multiple studies have demonstrated the frequent occurrence of mitochondrial impairment in various cancers.^243^, ^247^, ^248^ However, aerobic glycolysis is also observed in well‐functioning mitochondria in cancer cells in the presence of oxygen. This finding shows that the hypothesis offered by Warburg lacks persuasion.^249^, ^250^, ^251^ Cancer cells exhibit alterations in the genes associated with glucose metabolism, including well‐characterized mutations in RAS and MYC oncogenes, dysregulation of PI3K/AKT/mTOR and LKB1/AMPK signaling pathways, and the deletion of the tumor suppressor p53.^252^, ^253^ Emerging evidence has revealed that alterations in HIF‐1α, oncogenes, and tumor suppressor genes lead to upregulated expression of GLUTs and key metabolic enzymes of glucose metabolism, thereby increasing the rate of glucose metabolism in various cancer types, including lung, breast, liver, and oral cancer.^254^, ^255^ For example, MYC, the major regulator of cell growth, enhances the expression of diverse transporters and enzymes during glycolysis, including GLUT1, hexokinase 2 (HK2), pyruvate dehydrogenase kinase isozyme 1 (PDK1), and lactate dehydrogenase A (LDHA).^256^, ^257^, ^258^ The tumor suppressor p53, which holds prominence, has also been demonstrated to upregulate the glycolytic pathway and disrupt the equilibrium between anabolism and redox interactions.^259^ It is generally acknowledged that glucose transport across the plasma membrane, facilitated by GLUTs, is the first rate‐limiting stage in glucose utilization.^260^, ^261^, ^262^ Multiple studies have reported GLUT1 upregulation owing to mutations in HIF‐1α, RAS, p53, and the PI3K/AKT signaling pathway in breast cancer (BC), esophageal cancer (EC), pancreatic cancer, and colorectal cancer (CRC). Moreover, the activity of the three critical enzymes of glycolysis, namely HK2, phosphofructokinase 1, and pyruvate kinases type M2 (PKM2), has also been found to be increased as a result of the activation of AMPK, HIF‐1α, MYC, and the PI3K/AKT signaling pathway.^263^, ^264^ Most notably, somatic mutations of IDH, succinate dehydrogenase (SDH), and FH, which are enzymes involved in glucose metabolism, also cause metabolic perturbations by altering metabolite levels.^265^, ^266^, ^267^, ^268^, ^269^ Certain metabolites, including acetyl‐coenzyme A (acetyl‐CoA) and α‐ketoglutarate (α‐KG) also participate in the epigenetic reprogramming of glucose metabolism.^213^, ^270^, ^271^ More importantly, the cooccurrence of excessively activated mTOR signaling pathways and microenvironmental stimuli has been observed to lead to a novel glycolytic phenotype in hematological malignancies.^272^

#### 4.1.2 Gln metabolism

Besides increased glucose metabolism, cancer cells also exhibit substantial uptake of amino acids to sustained proliferation and the biosynthesis of biological macromolecules.^273^ Gln serves as not only a carbon substrate that enters the TCA cycle to fuse with biosynthetic precursors, including acetyl‐CoA and oxaloacetate, and as a bioenergetic source for NADH and FADH2, but also a nitrogen substrate that is metabolized to address the rapidly increasing demand for the synthesis of nucleotides, glucosamine‐6‐phosphate, asparagine, and even NAD in cancer cells.^274^ In 1935, Hans Krebs, the individual credited for discovering the TCA cycle, demonstrated the significance of Gln metabolism in the maintenance of organismal homeostasis using animal models, thereby sparking an interest among researchers to investigate the role of Gln in cell growth.^275^ In the 1950s, the American physiologist Harry Eagle was the first to propose that proliferating cancer cells exhibit an increased requirement for Gln based on the observation that human carcinoma cells (HeLA strain) exhibit higher uptake of Gln than other amino acids.^276^ Gln, the most abundant nonessential amino acid in the plasma and tissues, is the most indispensable nutrient besides glucose for cancer metabolism in various cancer types, including pancreatic ductal adenocarcinoma (PDAC), AML, colon cancer, PCa, kidney cancer, lung cancer, liver cancer, and glioma.^277^, ^278^, ^279^ On the one hand, amino acids are metabolized to meet the increased requirements for protein synthesis in proliferating cancer cells. On the other hand, the requirement for the catabolism of amino acids, especially Gln, for energy generation and biomass synthesis surpasses that for protein synthesis in cancer cells.^280^, ^281^ Indeed, cancer cells are profoundly dependent on Gln metabolism, a phenomenon commonly referred to as Gln addiction.^282^ Cancer cells inevitably experience a deficit of nutrients and energy; therefore, they frequently exhibit aberrant activation of certain genes and signaling pathways to address the increased demands for nutrients and energy.^283^ The expression of key enzymes involved in Gln metabolism has been found to be upregulated owing to onco‐genotypes and the TME. The aberrant activation of oncogenic factors and loss of tumor suppressors, such as MYC, RAS, p53, PTEN, and mTOR, has been implicated in Gln metabolism reprogramming.^275^, ^284^, ^285^, ^286^, ^287^, ^288^ Recent research has shown that MYC upregulates the expression of the Gln transporters alanine–serine–cysteine transporter 2 (ASCT2),^289^, ^290^, ^291^ which facilitates the uptake of Gln, and glutaminase (GLS), which facilitates the transformation of Gln to glutamate and subsequently α‐KG, thereby promoting the TCA cycle.^292^, ^293^ The loss of the tumor suppressor Rb also leads to increased Gln uptake by upregulating the expression of ASCT2 and GLS1. Moreover, MYC increases the expression of the enzymes involved in nucleotide biosynthesis, such as carbamoyl phosphate synthetase II (CAD), phosphoribosyl pyrophosphate synthetase 2, thymidylate synthase, and inosine monophosphate dehydrogenase.^280^, ^294^ Additionally, mutant RAS drives micropinocytosis to degrade extracellular proteins by remodeling the actin cytoskeleton for the supplement of amino acids, including Gln.^295^, ^296^, ^297^ KAS facilitates the crucial metabolism of the Gln carbon skeleton by upregulating aspartate transaminase (GOT) expression and thus maintains equilibrium between redox balance and the increasing growth demand of PDAC cells.^298^ Gln metabolism is regulated by the mTOR pathway, which induces glutamate dehydrogenase expression to convert glutamate to α‐KG.^286^, ^299^ Moreover, mTOR also upregulates CAD, thereby facilitating Gln to participate in nucleotide synthesis.^300^, ^301^ Interestingly enough, the products of Gln metabolism, such as glutamate, α‐KG, and aspartate, and even Gln itself have been observed to influence Gln metabolism.^275^, ^302^, ^303^ Furthermore, Gln metabolism in cancer cells has been observed to be also affected by conditions in the TME, especially hypoxia.^304^ A unique Gln metabolic phenotype is observed in lung cancer and liver cancer, which underscores the importance of the TME in cancer metabolic reprogramming.^230^ Adaptation to hypoxia has been found to typically lead to HIF‐1α overexpression and mediate Gln catabolism through the downregulation of MYC expression in diverse cancer types, including glioma, CC, EC, BC, and ovarian cancer.^231^, ^305^, ^306^, ^307^ Increased levels of reactive oxygen species (ROS) in the TME upregulate HIF‐1α expression, and glutathione generated from Gln downregulates HIF‐1α expression.^308^, ^309^ These insights into Gln metabolism have led to ongoing research efforts aimed at understanding how targeting Gln utilization in cancer cells could potentially yield therapeutic benefits.

#### 4.1.3 Lipid metabolism

Studies have indicated that lipids supply energy and biomass via FA oxidation (FAO), contribute to the formation of cell membranes, maintain cell barrier function and fluidity, and participate in intercellular communication and various biological processes during growth and development.^310^, ^311^ Multiple studies have demonstrated substantial alterations in lipid metabolism, particularly FA synthesis, across various cancer types.^312^, ^313^ Cancer cells commonly exhibit elevated de novo lipogenesis or lipid uptake to sustain rapid proliferation, adapt to changes in the surrounding environment, and produce signaling molecules.^314^ Several studies have shown that in various cancer types, altered lipid metabolism contributes to the migration of cancer cells, the promotion of angiogenesis, the evasion of immune surveillance, and drug resistance.^315^, ^316^, ^317^ It is well established that multiple oncogenic signaling pathways and transcription factors lead to the overexpression of key enzymes in FA synthesis.^318^ In cancer cells, constitutively activated AKT has been found to downregulate the level of sterol regulatory element binding protein (SREBP) under the synergy of mTORC1 and subsequently enhance de novo lipogenesis.^319^, ^320^ High MYC expression in cancer cells also upregulates the transcriptional activator MondoA, thereby promoting lipid biosynthesis.^321^ Additionally, numerous studies have validated that lipid metabolism is susceptible to the changes in TME. Under conditions of hypoxia, reduced FA synthesis leads cancer cells to increase exogenous lipid uptake, modify cellular lipids, and scavenge lysophospholipids.^322^

#### 4.1.4 Other metabolisms

Despite the typical increase in glycolytic rates in cancer cells, most cancer cells generate the majority of ATP through mitochondrial function. Mitochondria, as cellular energy factories, mediate metabolism, bioenergetics, redox homeostasis, and ROS in cells through OXPHOS.^323^ The mitochondrial TCA cycle has been found to participate in DNA modifications and posttranslational modifications of histones.^324^, ^325^, ^326^ Emerging evidence indicates that genetic alterations of multiple mitochondrial metabolic enzymes, including IDH1/2, SDH, and FH, drive tumorigenesis in diverse cancers.^327^, ^328^, ^329^, ^330^ For example, SDH type B (SDHB)‐mutated pheochromocytomas/paragangliomas tend to exhibit more invasive phenotypes.^331^ Gain‐of‐function mutations in IDH1/2 generate excess oncometabolite D‐2‐hydroxyglutarate, which leads to metabolic changes in chromatin structure and contributes to tumorigenesis.^332^, ^333^ Moreover, the accumulation of certain mitochondrial metabolites in the TME has been found to contribute to cancer progression.^334^ Increased concentrations of 2‐HG contribute to multiple malignant processes and signal transduction in cancer cells.^335^, ^336^, ^337^ Mounting evidence suggests that succinate accumulation directly induces oncogenic effects in diverse cancer types, primarily through epigenetic modifications and the activation of hypoxic signaling.^338^ Notably, mitochondrial metabolic reprogramming is a dynamic process accompanied by energy requirements of cell growth during various stages of cancer development, suggesting that mitochondrial metabolism is a double‐edged sword in tumorigenesis.^339^, ^340^ For example, ROS produced as a result of mitochondrial metabolism has dual functions within cells. Elevated levels of ROS lead to oxidative damage of cellular proteins, lipids, and nucleic acids, and even cell death. A chronic increase in ROS levels below a certain threshold enhances malignant behavior of cancer cells.^323^, ^341^ Accumulating evidence emphasizes the characteristics of mitochondrial metabolism in cancer progression, thereby driving investigation into mitochondrial metabolism.^342^, ^343^, ^344^

### 4.2 Association of RNA methylation with abnormal cancer metabolism

A growing body of evidence suggests that RNA methylation is broadly dysregulated in human cancer and has been implicated in the regulation of cancer metabolic reprogramming.^345^ The dysregulation of glucose, lipid, and Gln metabolism is closely associated with malignant bioprocesses, including cell proliferation, invasion, and drug resistance. Cancer metabolic reprogramming involves alterations in the expression of various metabolism‐associated proteins and downstream signaling pathways. Recent studies have demonstrated that RNA methylation is closely associated with the metabolic reprogramming of cancer cells. In cancer, the specific functions of RNA methylation primarily depend on the category of targeted genes (oncogenic or tumor‐suppressive effect), the activity of writers and erasers, and the regulation of readers. In addition, distinct metabolic pathways are likely regulated by the same signaling pathways and methylation‐modified RNA.^26^ Most notably, RNA modification plays a paradoxical role in cancer, wherein certain genes, upon methylation, promote cancer development, whereas others impede cancer progression.^346^ In the subsequent sections, we emphatically discuss the recent discoveries concerning the effect of commonly observed RNA methylation modifications on cancer metabolism, including glucose, amino acid, and FA metabolism. We also summarize the mechanisms underlying RNA methylation concerning cancer metabolic reprogramming (Table 1).

**TABLE 1:** Role of RNA methylation in cancer metabolism reprogramming.

Metabolic patterns	RNA‐modified regulators	RNA‐modified targets	Related molecules or pathways	Functional effects	Corresponding diseases	Property	References
Glucose metabolism	METTL3 and IGF2BP2/3	HK2	GLUT1	Aerobic glycolysis ↑	CRC	Oncogene	^352^
	IGF2BP2	lncRNA LINRIS	MYC	Aerobic glycolysis ↑	CRC	Oncogene	^355^
	METTL3	GLUT1	mTORC1 signaling pathway	Aerobic glycolysis ↑	CRC	Oncogene	^349^
	IGF2BP2	lncRNA ZFAS1	OLA1	Warburg effect ↑, cell proliferation ↑, colony formation ↑, and apoptosis ↓	CRC	Oncogene	^358^
	METTL3 and IGF2BP2	PTTG3P	/	Glycolysis ↑ and cell proliferation ↑	CRC	Oncogene	^354^
	METTL3 and YTHDF1	JAK1	JAK1–STAT3 signaling	Lactate generation ↑ and immunosuppression ↑	CRC	Oncogene	^353^
	METTL3	LDHA	/	Glucose consumption ↑, lactate generation ↑, ATP generation ↑, and oxygen consumption rate ↑	CRC	Oncogene	^350^
	YTHDF2	circ‐0003215	miR‐663b/DLG4/G6PD axis	Pentose phosphate pathway ↓, cell proliferation ↓, migration ↓, and invasion ↓	CRC	Tumor suppressor	^357^
	KIAA1429	HK2	/	Aerobic glycolysis ↑, glucose uptake ↑, lactate production ↑, ATP generation ↑, and extracellular acidification rate ↑	CRC	Oncogene	^356^
	METTL3	LINC01615	G6PD	Pentose phosphate pathway ↑	CRC	Oncogene	^351^
	WTAP	NT5DC3	HKDC1	Tumor growth under hyperglycemia↓	CRC	Tumor suppressor	^359^
	METTL3	/	mTOR signaling pathway	Aerobic glycolysis ↑	HCC	Oncogene	^360^
	METTL3	HIF‐1α	HBXIP	Warburg effect ↑	HCC	Oncogene	^361^
	YTHDF1 and IGF2BP3	PDK4	/	Glycolysis ↑	HCC	Oncogene	^364^
	YTHDF3	PFKL	/	Glycolysis ↑	HCC	Oncogene	^365^
	IGF2BP2	mir4458hG	HK2 and GLUT1	Glycolysis ↑, cell proliferation ↑, and macrophage polarization↑	HCC	Oncogene	^366^
	YTHDF1	circRHBDD1	PIK3R1	Aerobic glycolysis ↑	HCC	Oncogene	^363^
	METTL3 and IGF2BP2	WWP2	AKT signaling pathway	Aerobic glycolysis ↑	HCC	Oncogene	^362^
	ALKBH5	UBR7	Keap1/Nrf2/Bach1/HK2	Aerobic glycolysis ↑	HCC	Tumor suppressor	^368^
	METTL14	USP48	SIRT6	Glycolysis ↓	HCC	Tumor suppressor	^369^
	ZC3H13	PKM2	/	Glycolysis and cisplatin sensitization	HCC	Suppressor	^367^
	METTL14	LHPP	GSK3β and HIF‐1α	Glycolysis ↑, cell proliferation ↑, invasion ↑, and metastasis ↑	GC	Oncogene	^377^
	METTL3 and IGF2BP3	HDGF	GLUT4 and ENO2	Aerobic glycolysis ↑, proliferation ↑, and liver metastases ↑	GC	Oncogene	^370^
	METTL3 and IGF2BP1	NDUFA4	/	Glycolysis ↑ and cell proliferation ↑	GC	Oncogene	^371^
	WTAP	HK2	/	Glycolysis ↑, and cell proliferation ↑	GC	Oncogene	^376^
	KIAA1429	LINC00958	GLUT1	Aerobic glycolysis ↑	GC	Oncogene	^375^
	FTO and YTHDF2	PRKAA1	/	Glycolysis ↑ and cell proliferation ↑	GC	Oncogene	^374^
	IGF2BP1	c‐MYC	/	Glycolysis ↑ and cell proliferation ↑	GC	Oncogene	^373^
	IGF2BP3	c‐MYC	LOC101929709, LIN28B, and PI3K/AKT pathway	Glycolysis ↑, cell proliferation ↑, proliferation ↑, and migration↑	GC	Oncogene	^372^
	YTHDC1	miR‐30d	RUNX1, GLUT1, and HK1	Aerobic glycolysis ↓, tumor growth ↓, and metastasis ↓	PDAC	Tumor suppressor	^378^
	IGF2BP2	GLUT1	/	Aerobic glycolysis ↑ and cell proliferation ↑	PDAC	Oncogene	^379^
	ALKBH5	GLUT4	/	Aerobic glycolysis ↑	Breast cancer	Oncogene	^383^
	METTL3 and YTHDF2	LATS1	Hippo pathway	Aerobic glycolysis ↑	Breast cancer	Oncogene	^380^
	WTAP	ENO1	ERK1/2 signaling pathway	Glycolysis ↑	Breast cancer	Oncogene	^381^
	YTHDF1	PKM2	HIF‐1α and miR‐16‐5p	Glycolysis ↑, cell proliferation ↑, and invasion ↑	Breast cancer	Oncogene	^382^
	METTL3 and YTHDF1	HK2	/	Aerobic glycolysis ↑ and cell proliferation ↑	Cervical cancer	Oncogene	^384^
	IGF2BP2	MYC	HPV E6/E7	Aerobic glycolysis ↑	Cervical cancer	Oncogene	^385^
	FTO	HK2	HPV E6E7 and GSK3β	Aerobic glycolysis ↓	Cervical cancer	Tumor suppressor	^386^
	ALKBH5	CK2α	/	Glycolysis ↓, cell proliferation ↓, migration ↓, invasion ↓, cisplatin chemosensitivity ↑, and apoptosis ↑	Bladder Cancer	Tumor suppressor	^388^
	METTL3	lncRNA SNHG7	SRSF1 and c‐Myc	Glycolysis ↑ and cell proliferation ↑	Prostate cancer	Oncogene	^389^
	METTL14	BPTF	/	Glycolysis ↓	RCC	Tumor suppressor	^387^
	FTO and YTHDF2	PFKP and LDHB	R‐2HG	Glycolysis ↑	AML	Oncogene	^390^
	FTO and IGF2BP2	APOE	IL‐6/JAK2/STAT3 signaling pathway	Glycolysis ↓ and cell growth ↓	Papillary thyroid cancer	Tumor suppressor	^391^
	METTL3 and YTHDF	APC	Wnt/β‐catenin pathway, cyclin D1, c‐Myc, and PKM2	Aerobic glycolysis ↑, cell proliferation ↑, and tumor formation ↑	ESCC	Oncogene	^392^
	FTO and YTHDF1	c‐Myc	Wnt/β‐catenin pathway	Glycolysis ↓ and cell proliferation ↓	Lung adenocarcinoma	Tumor suppressor	^393^
	FTO	PDK1	lncRNA JPX	Aerobic glycolysis ↑ and temozolomide chemoresistance ↑	Glioblastoma	Oncogene	^394^
	ALYREF	PKM2	HIF‐1α	Aerobic glycolysis ↑ and cell proliferation ↑	Bladder cancer	Oncogene	^395^
	ALKBH3 and YTHDF1	ATP5D	E2F1	Aerobic glycolysis ↑	Cervical cancer	Oncogene	^397^
Glutamine metabolism	YTHDF1	GLS1	/	Glutamine metabolism ↑ and cisplatin resistance ↑	CRC	Oncogene	^399^
	FTO and YTHDF2	ATF4	DDIT4 and mTOR signal pathway	Prosurvival autophagy under glutaminolysis inhibition↑	CRC	Oncogene	^400^
	FTO	SLC1A5	/	Glutamine metabolism ↑, cell growth↑, and survival↑	RCC	Oncogene	^401^
	METTL14	CSAD, GOT2, and SOCS2	**/**	Glutamine metabolism ↑ and cisplatin resistance ↑	HCC	Tumor suppressor	^402^
	IGF2BP2	MYC, GPT2, and SLC1A5	/	Glutamine metabolism ↑	AML	Oncogene	^398^
Lipid metabolism	METTL3 and METTL14	ACLY	SCD1	Lipogenesis ↑	HCC	Oncogene	^403^
	METTL5 and TRMT112	ACSL	/	Fatty acid metabolism ↑	HCC	Oncogene	^404^
	FTO	Triglyceride	/	Lipid metabolism ↑ and triglyceride deposition ↑	HCC	/	^409^
	METTL3	LINC00958	/	Lipogenesis ↑	HCC	Oncogene	^406^
	METTL3 and YTHDF1	SLP2	JNK2 and SREBP1	De novo lipogenesis ↑, cell proliferation ↑, and metastasis ↑	HCC	Oncogene	^405^
	FTO	FASN	/	Lipid metabolism ↑ and cell apoptosis ↓	HCC	Oncogene	^407^
	FTO	SREBP1c and CIDEC	/	Lipogenesis ↑	HCC	Oncogene	^408^
	METTL3	ERRγ	ABCB1 and CPT1B	Fatty acid oxidation **↑**	HCC	Oncogene	^410^
	FTO and YTHDF1	HSD17B11	**/**	Lipid metabolism ↑, cell proliferation ↑, invasion ↑, stemness ↑, and tumorigenicity ↑	EC	Oncogene	^412^
	ALKBH5 and HNRNPA2B1	ACLY and ACC1	/	De novo lipogenesis ↑, cell proliferation ↑, migration ↑, and invasion ↑	EC	Oncogene	^413^
	METTL3 and YTHDF2	DEGS2	/	Ceramide synthesis ↑, cell proliferation ↓, and migration ↓,	CRC	Tumor suppressor	^411^
	IGF2BP2	PRMT6	MFSD2A	Docosahexaenoic acid A in the plasma ↑ and stem cell maintenance ↑	AML	Oncogene	^414^
	IGF2BP1	ALKBH5	SIRT3 and ACC1	Lipid metabolism ↓	Cervical cancer	Tumor suppressor	^415^
	METTL14	LncDBET	FABP5 and PPAR signaling pathway	Lipid metabolism ↑	Bladder cancer	Oncogene	^416^
	YTHDF2	LXRA and HIVEP2	EGFR/SRC/ERK signaling pathway	Cholesterol homeostasis ↓, cell proliferation ↑, invasion ↑, and tumorigenesis ↑	Glioblastoma	Oncogene	^417^
	NSUN2	FABP5	/	Lipid metabolism ↑, cell proliferation ↑, invasion ↑, and migration ↑	Osteosarcoma	Oncogene	^420^
Mitochondrial metabolism	FTO	Caveolin‐1	/	Mitochondrial metabolism ↑, cell proliferation ↑, and metastasis ↑	GC	Oncogene	^423^
	METTL3 and IGF2BP1	NDUFA4	/	Mitochondrial fission ↑, ROS production ↓, and proliferation ↑	GC	Oncogene	^371^
	METTL3	HIF‐2α	MTHFD2	Mitochondrial one‐carbon metabolism ↑ and glycolysis ↑	RCC	Oncogene	^424^
	FTO	PGC‐1α	/	Mitochondrial activity ↑, oxidative stress ↑, ROS production ↑, and tumor growth ↓	RCC	Tumor suppressor	^425^
	METTL3	RALY	/	Mitochondrial respiration ↓, ROS production ↓, cell proliferation ↑, apoptosis ↑, and metastasis ↑	CRC	Oncogene	^422^
	METTL3	AK4	/	Mitochondrial apoptosis ↓ and resistance to tamoxifen ↓	Breast cancer	Tumor suppressor	^426^
	ALKBH7	mt‐tRNA	/	Mitochondrial activity↑ and OXPHOS function↑	HCC	/	^151^

HK2, hexokinase 2; GLUT1, glutamate dehydrogenase 1; OLA1, Obg‐like ATPase 1; PTTG3P, pituitary tumor‐transforming gene 3 pseudogene; LDHA, lactate dehydrogenase A; DLG4, discs large MAGUK scaffold protein 4; G6PD, glucose‐6‐phosphate dehydrogenase; NT5DC3, 5′‐nucleotidase domain‐containing protein 3; HKdc1, hexokinase domain component 1; HBXIP, Hepatitis B virus X‐interacting protein; PDK4, pyruvate dehydrogenase kinase isozyme4; PFKL, phosphofructokinase, liver type; WWP2, WW domain‐containing protein 2;UBR7, ubiquitin protein ligase E3 component N‐recognin 7; Keap1, kelch‐like ECH‐associated protein 1; Nrf2, nuclear factor erythroid 2‐related factor 2; Bach1, BTB domain and CNC homolog 1; USP48, ubiquitin‐specific peptidase 48; SIRT6, sirtuin 6; PKM2, pyruvate kinases type M2; LHPP, phospholysine phosphohistidine inorganic pyrophosphate phosphatase; GSK3β, glycogen synthase kinase‐3β; HDGF, hepatoma‐derived growth factor; ENO2, enolase 2; NDUFA4, NADH dehydrogenase (ubiquinone) 1 alpha subcomplex 4; PRKAA1, AMPK catalytic subunit α1; RUNX1, Runt‐related transcription factor 1; LATS1, large tumor suppressor kinase 1; ERK1/2, extracellular signal‐regulated kinase 1/2; CK2α, casein kinase 2α; SRSF1, serine/arginine‐rich splicing factor 1; BPTF, bromodomain PHD finger transcription factor; PFKP, phosphofructokinase platelet; LDHB, lactate dehydrogenase B; R‐2HG, R‐2‐hydroxyglutarate; APOE, apolipoprotein E; APC, ATP5D, the δ subunit of ATP synthase; E2F1, E2F transcription factor 1; LXRα, liver X receptor alpha; GLS1, glutaminase 1; ATF4, activating transcription factor 4; DDIT4, DNA damage‐inducible transcript 4; SLC1A5, solute carrier family 1 member 5; CSAD, cysteine sulfinic acid decarboxylase; GOT2, glutamic‐oxaloacetic transaminase 2; GPT2, glutamic pyruvate transaminase 2; SLC1A5, solute carrier family 1 member 5; ACLY, ATP citrate lyase; SCD1, stearoyl‐CoA desaturase 1; ACSL, acyl‐CoA synthetase long‐chain family; SLP2, stomatin‐like protein 2; JNK2, c‐Jun N‐terminal kinase 2; SREBP1, sterol regulatory element binding protein‐1; FASN, FA synthase; CIDEC, cell death‐inducing DFFA (DNA fragmentation factor‐α)‐like effector c; ERRγ, estrogen receptor‐related receptor γ; ABCB1, ATP binding cassette subfamily B member 1; CPT1B, carnitine palmitoyltransferase 1B; HSD17B11, 17beta‐hydroxysteroid dehydrogenase 11; ACC1, acetyl‐CoA carboxylase 1; DEGS2, delta(4)‐desaturase, sphingolipid 2; PRMT6, protein arginine methyltransferase 6; MFSD2A, major facilitator superfamily domain containing 2A; SIRT3, silent mating type information regulation 2 homologue 3; FABP5, FA binding protein 5; LXRα, liver X receptor alpha; HIVEP2, human immunodeficiency virus type I enhancer binding protein 2; FABP5, FA binding protein 5; NDUFA4, NADH dehydrogenase (ubiquinone) 1 alpha subcomplex 4; MTHFD2, PGC‐1α, PPARδ coactivators‐1α; RALY, AK4, adenylate kinase 4; CRC, colorectal cancer; HCC, hepatocellular carcinoma; GC, Gastric cancer; PDAC, pancreatic ductal adenocarcinoma; RCC, renal cell carcinoma; AML, acute myeloid leukemia; ESCC, esophageal squamous cell carcinoma; EC, EC.

#### 4.2.1 Glucose metabolism and RNA methylation in cancer

**FIGURE 3:**
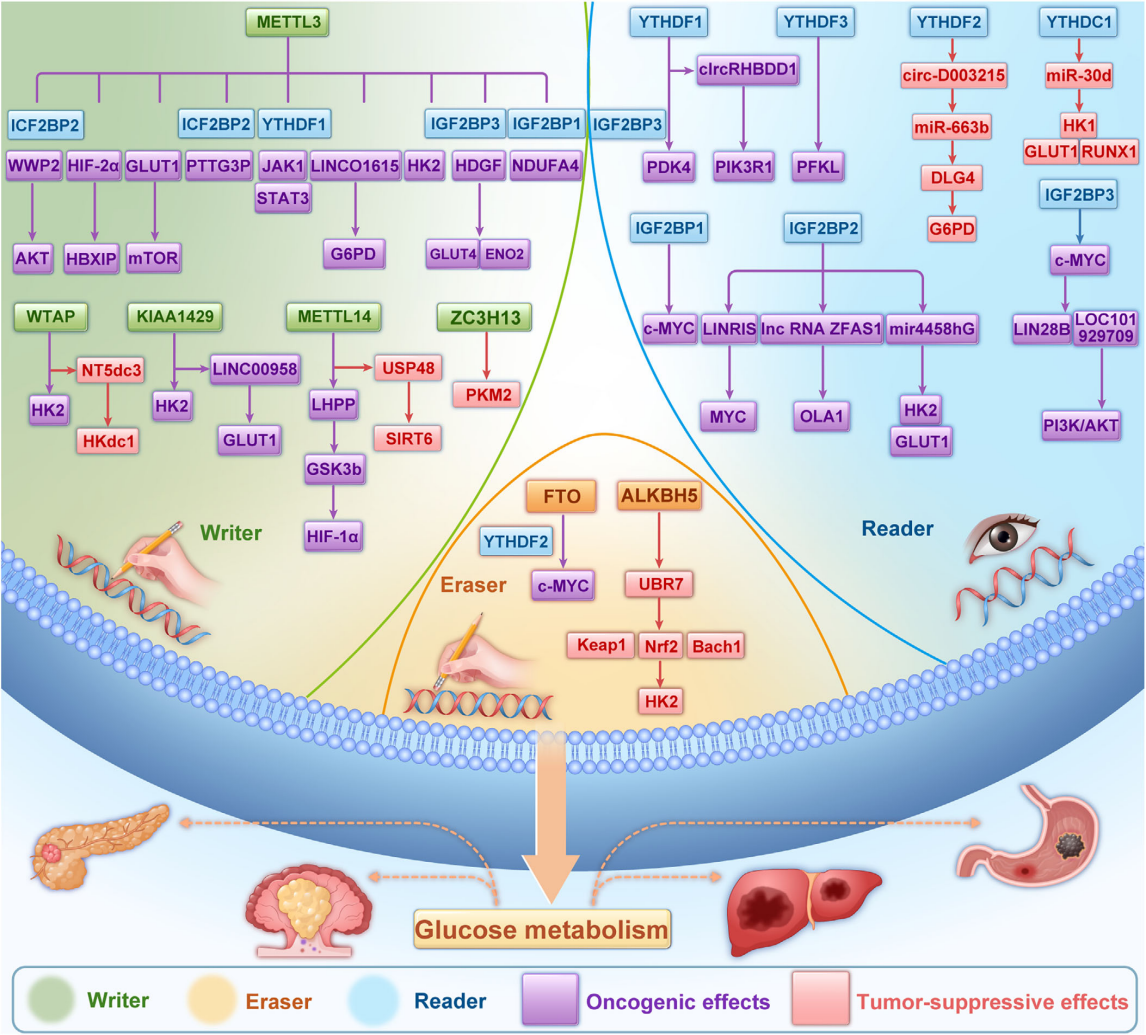
m6A methylation exerts core effects on glucose metabolism for cancer progression in digestive system cancers. In digestive system cancers, m^6^A methylation regulates the expression of downstream molecules to disrupt aerobic glucose metabolism in cancer cells, thereby contributing to cancer progression. The dynamic regulation of m^6^A modification is primarily performed by the m^6^A methyltransferases (writers), demethylases (erasers), and m^6^A‐binding proteins (readers). Oncogenic or tumor‐suppressive functions and the expression of these m^6^A regulators differ across multiple cancers. Various m^6^A readers preferentially recognize m^6^A‐modified RNAs to regulate their stability and expression. m6A writers and erasers also modify RNAs associated with glucose metabolism, thereby affecting tumorigenesis in several types of cancer, including colorectal cancer, liver cancer, gastric cancer, and pancreatic cancer. The purple boxes represent oncogenic effects, and the pink boxes represent tumor‐suppressive effects. PTTG3P, pituitary tumor‐transforming gene 3 pseudogene; GLUT1, glutamate dehydrogenase 1; OLA1, Obg‐like ATPase 1; DLG4, discs large MAGUK scaffold protein 4; G6PD, glucose‐6‐phosphate dehydrogenase; HBXIP, Hepatitis B virus X‐interacting protein; WWP2, WW domain‐containing protein 2; UBR7, ubiquitin protein ligase E3 component N‐recognin 7; Keap1, kelch‐like ECH‐associated protein 1; Nrf2, nuclear factor erythroid 2‐related factor 2; Bach1, BTB domain and CNC homolog 1; USP48, ubiquitin‐specific peptidase 48; SIRT6, sirtuin 6; PKM2, pyruvate kinases type M2; GSK3β, glycogen synthase kinase‐3β; HDGF, hepatoma‐derived growth factor; ENO2, enolase 2; NDUFA4, NADH dehydrogenase (ubiquinone) 1 alpha subcomplex 4; RUNX1, Runt‐related transcription factor 1.

Accumulating evidence shows that RNA methylation, especially m^6^A RNA methylation, plays a vital role in the modulation of glucose metabolism in multiple human cancers.^346^ In digestive system cancers, RNA methylation is dysregulated and participates in the activation of the glycolytic pathway (Figure 3).^347^, ^348^ In multiple cohorts of patients with CRC, the overexpression of the m^6^A writer METTL3 has been validated to be correlated with poor prognosis.^24^ METTL3 upregulates GLUT1, thereby increasing glucose uptake and lactate production, which in turn activates mTORC1 signaling to promote CRC development.^349^ In 5‐fluorouracil (5‐FU)‐resistant CRC cells, METTL3 elevates LDHA translation, thereby enhancing glycolysis and 5‐FU chemoresistance.^350^ METTL3 induces the upregulation of LINC01615, thereby increasing glucose‐6‐phosphate dehydrogenase (G6PD) expression, increasing the PPP flux, sustaining cell survival, and modulating oxaliplatin chemosensitivity under conditions of nutrient deprivation.^351^ Knockout of METTL3 in xenograft mouse tumor model also exhibits the impaired glucose uptake and inhibited tumor growth. METTL3 has been observed to upregulate and maintain GLUT1 and HK2 expression. This leads to upregulated glucose metabolism through an m^6^A reader IGF2BP2/3‐dependent pattern during CRC tumorigenesis, respectively.^352^ Additionally, H3K18 lactylation induced by lactate accumulation upregulates METTL3 expression and subsequently mediates m^6^A modification of JAK1, thereby enhancing the immunosuppressive roles of CRC tumor‐infiltrating myeloid cells.^353^, ^354^ Studies have also elucidated that LINRIS inhibits IGF2BP2 degradation in CRC HCT116 cells by inhibiting K139 ubiquitination, thereby promoting downstream MYC‐mediated glycolysis and tumor proliferation. In vivo experiment on the BALB/c nude mice injected HCT116 cells with or without LINRIS knockdown observes that LINRIS knockdown suppresses the growth of CRC tumors and glycolysis mediated by IGF2BP2–MYC axis.^355^ Emerging evidence indicates that the m^6^A methyltransferase KIAA1429 promotes aerobic glycolysis by upregulating HK2 expression.^356^ Moreover, the m^6^A writer YTHDF2 degrades circ_0003215 to suppress signaling via the miR‐663b/discs large MAGUK scaffold protein 4 (DLG4)/G6PD axis and subsequently upregulate PPP and enhance the malignant phenotypes of CRC.^357^ And IGF2BP2 also enhances lncRNA ZFAS1 expression to elevate the activity of Obg‐like ATPase 1 (OLA1) and ultimately accelerates glycolysis and CRC progression.^358^ Remarkably, Lactoferrin was recently found to alleviate the effect of the m^6^A writer WTAP on 5′‐nucleotidase domain‐containing protein 3 (NT5DC3) and Hexokinase domain component 1 (HKDC1) expression to suppress CRC progression under conditions of hyperglycemia.^359^ In HCC, upregulated METTL3 expression is correlated with poor outcomes and has been validated to upregulate glycolysis by promoting mTOR activity.^360^ Hepatitis B virus X‐interacting protein (HBXIP) positively modulates METTL3 level to interfere with the m^6^A methylation of HIF‐1α, thereby regulating the rate of aerobic glycolysis and the malignant behavior of HCC cells.^361^ In clinical samples of HCC samples and HCC cells, METTL3 and IGF2BP2 have been shown to induce the m^6^A modification of WW domain‐containing protein 2 (WWP2), thereby upregulating WWP2 expression, activating AKT signaling, and enhancing glycolysis and doxorubicin resistance.^362^ Moreover, the circRNA circRHBDD1 upregulates YTHDF1 expression, thereby inducing PIK3R1 translation and attenuating the response to anti‐PD‐1 therapy.^363^ YTHDF1 also promotes PDK4 expression under the involvement of IGF2BP3 to enhance glycolysis and ATP generation in HCC.^364^ The m^6^A reader YTHDF3 suppresses the degradation of the phosphofructokinase (PFKL) and establishes a positive functional loop with PFKL to promote HCC progression through glucose metabolism.^365^ The lncRNA mir4458hG, along with IGF2BP2, upregulates HK2 and GLUT1 expression to upregulate glucose metabolism in HCC.^366^ In contrast, the m^6^A methyltransferase ZC3H13 impairs PKM2 mRNA stability, thereby attenuating glycolysis and improving the susceptibility of HCC cells to cisplatin.^367^ And investigation of clinical human samples has revealed that the m^6^A demethylase ALKBH5 upregulates the expression of ubiquitin protein ligase E3 component N‐recognin 7 (UBR7), which is a negative regulator of glycolysis, and activates the kelch‐like ECH‐associated protein 1 (Keap1)/nuclear factor erythroid 2‐related factor 2 (Nrf2)/BTB domain and CNC homolog 1(Bach1)/HK2 pathway, thereby attenuating aerobic glycolysis in HCC.^368^ METTL14 has also been reported to upregulate the expression of the ubiquitin‐specific peptidase 48 (USP48) and the histone deacetylase sirtuins (SIRT6), thereby performing a suppressive function in the metabolic reprogramming of HCC cells.^369^ Gastric cancer (GC) exhibits increased levels of m^6^A‐modified RNA, which shows a strong association with poor clinical outcomes in patients. Stimulation of METTL3 by P300‐mediated H3K27 acetylation and its subsequent cooperation with IGF2BP3 upregulates HDGF, whereas its collaboration with IGF2BP1 upregulates NADH dehydrogenase (ubiquinone) 1 alpha subcomplex 4 (NDUFA4) expression, which is associated with the upregulation of glycolysis in GC.^370^, ^371^ LIN28B collaborates with IGF2BP3 to recognize and stabilize c‐MYC transcription and promotes aerobic glycolysis in the presence of LOC101929709.^372^ In vitro assays have revealed that IGF2BP1 directly interacts with c‐MYC to upregulate aerobic glycolysis in GC cells.^373^ FTO regulates the m^6^A modification of AMPK catalytic subunit α1 (PRKAA1) and enhances the ability of PRKAA1 to promote glycolysis in GC.^374^ The lncRNA LINC00958 is modified by the m^6^A methyltransferase KIAA1429 and interacts with GLUT1 to induce aerobic glycolysis in GC.^375^ Functional experiments have shown that WTAP bound to the 3′‐UTR m^6^A site of HK2 enhances the glycolytic capacity of GC cells.^376^ Additionally, METTL14 increases the mRNA stability of the acetylated protein LHPP, thereby attenuating glycogen synthase kinase‐3β (GSK‐3β) phosphorylation and the Wnt signaling pathway to suppress aerobic glycolysis in GC cells.^377^ In PDAC, YTHDC1 augments miR‐30d expression to attenuate aerobic glycolysis by suppressing the activity of the transcription factor RUNX1 along with the promoters of SLC2A1 and HK1.^378^ Furthermore, the upregulation of IGF2BP2 has been reported to directly increase GLUT1 expression, thereby promoting aerobic glycolysis, which results in a poor prognosis for patients with PDAC.^379^

Similarly, RNA methylation is also involved in BC progression. METTL3 induces a high degree of m^6^A methylation of the tumor suppressor LATS1, which is further stabilized by YTHDF2, and attenuates the antitumor activity of the Hippo pathway, thereby promoting glycolysis and tumorigenesis.^380^ C5aR1‐positive neutrophils, a novel neutrophil subset, promote extracellular signal‐regulated kinase 1/2 (ERK1/2) signaling and stabilize WTAP to upregulate enolase 1 (ENO1) expression through the secretion of IL‐1β and TNF‐α, thereby promoting glycolysis in BC.^381^ The upregulated YTHDF1 has been observed to inhibit miR‐16‐5p expression under hypoxic conditions, thereby upregulating PKM2 expression to enhance glycolysis in BC cells.^382^ Furthermore, the m^6^A demethylase ALKBH5 upregulates GLUT4 expression in a YTHDF2‐dependent manner to promote glycolysis and resistance to HER2‐targeted therapy in BC cells.^383^ METTL3 is also upregulated in CC tissues and cells and is closely associated with the poor prognosis of patients. Functional analysis has shown that METTL3 pronounces the Warburg effect in CC cells by mobilizing YTHDF1 to stabilize HK2 expression.^384^ The upregulated expression of human papillomavirus (HPV) E6/E7 drives aerobic glycolysis in CC by directing IGF2BP2 to actively regulate MYC methylation or upregulate serine/threonine kinase GSK‐3β expression and by degrading m^6^A demethylase FTO to increase HK2 expression.^385^, ^386^ In renal cell carcinoma (RCC), METTL14 downregulates the expression of bromodomain PHD finger transcription factor (BPTF) to attenuate the oncogenic transcriptome, glycolytic reprogramming, and distal lung metastasis.^387^ In bladder cancer, the m^6^A demethylase ALKBH5 downregulates the expression of casein kinase 2α (CK2α) and glycolysis‐associated proteins to inhibit glycolysis and increase sensitivity of cancer cells to cisplatin.^388^ In PCa, METTL3 stabilizes the lncRNA SNHG7 to promote glycolysis via the serine/arginine‐rich splicing factor 1 (SRSF1)/c‐MYC axis.^389^ In leukemia, FTO and YTHDF2 are found to upregulate phosphofructokinase PFKP and LDHB levels to facilitate aerobic glycolysis, which process can be damped by a metabolite of mutant isocitrate dehydrogenases R‐2‐hydroxyglutarate (R‐2HG).^390^ In papillary thyroid cancer, FTO decreases the stability of apolipoprotein E mRNA through IGF2BP2‐induced m^6^A modification to abrogate glycolysis.^391^ In esophageal squamous cell carcinoma (ESCC), METTL3 and YTHDF collaborate to downregulate adenomatous polyposis coli (APC) expression, thereby activating the Wnt/β‐catenin signaling pathway and promoting aerobic glycolysis.^392^ In lung adenocarcinoma, Wnt/β‐catenin signaling, along with YTHDF1, inhibits FTO expression and subsequently enhances c‐MYC expression, thereby promoting aerobic glycolysis and malignant behavior in cancer cells.^393^ In glioblastoma multiforme (GBM), increased levels of the lncRNA that is proximal to X‐inactive (JPX) positively modulate the level of a limiting enzyme of glycolysis, namely PDK1, in an FTO‐dependent manner, thereby contributing to temozolomide chemoresistance.^394^

Besides the pathogenesis of the m^6^A modification in relation to glucose metabolism reprogramming in cancer, the m^5^C modification also affects glucose metabolism, thereby contributing to cancer development. Little is known about the specific biological function and roles of m^5^C methylation in cancer metabolism. A previous study revealed that the m^5^C modification plays a role in the carcinogenesis of bladder cancer by stabilizing oncogenic mRNAs, recovering the underlying mechanism of RNA m5C‐mediated oncogene activation.^178^ Furthermore, the m^5^C reader ALYREF has been reported to extend the half‐life of PKM2 mRNA, that is, stabilize PKM2 mRNA, by binding to the 3′‐UTR of the mRNA. HIF‐1α also indirectly upregulates PKM2 by cooperating with ALYREF to enhance glucose utilization, lactate production, and ATP generation, thereby increasing cell proliferation. These findings suggest that ALYREF significantly upregulates glycolysis and cell proliferation by stabilizing PKM2 mRNA in bladder cancer.^395^ In general, the function of m^1^A methylation in cancer development and cancer metabolic reprogramming has been largely unexplored. Emerging evidence demonstrates that the m^1^A methylation of RNA plays a role in the regulation of metabolic processes in cancer cells.^396^ A study revealed that the m^1^A eraser ALKBH3 targets exon 1 of ATP5D (the δ subunit of ATP synthase) mRNA and inhibits its transcription and translation through the YTHDF1/eRF3 axis. ALKBH3 knockdown has been shown to repress cell growth, migration, and invasion and decrease overall glycolytic flux. Furthermore, ATP5D overexpression upregulates glycolysis and ATP generation in cancer cells.^397^ The m^1^A eraser ALKBH1 notably induces the demethylation of targeted tRNAs, such as tRNA^Glu (CUC)^, tRNA^Lys (yUU)^, and tRNA^Gln (CUG)^, thereby rapidly responding to conditions of glucose deprivation.^158^ As considerable progress has been made in understanding the effects of RNA methylation on glucose metabolism in cancer circumstances, the regulatory mechanisms involved in their interaction can be exploited for clinical applications.

#### 4.2.2 Gln metabolism and RNA methylation in caner

**FIGURE 4:**
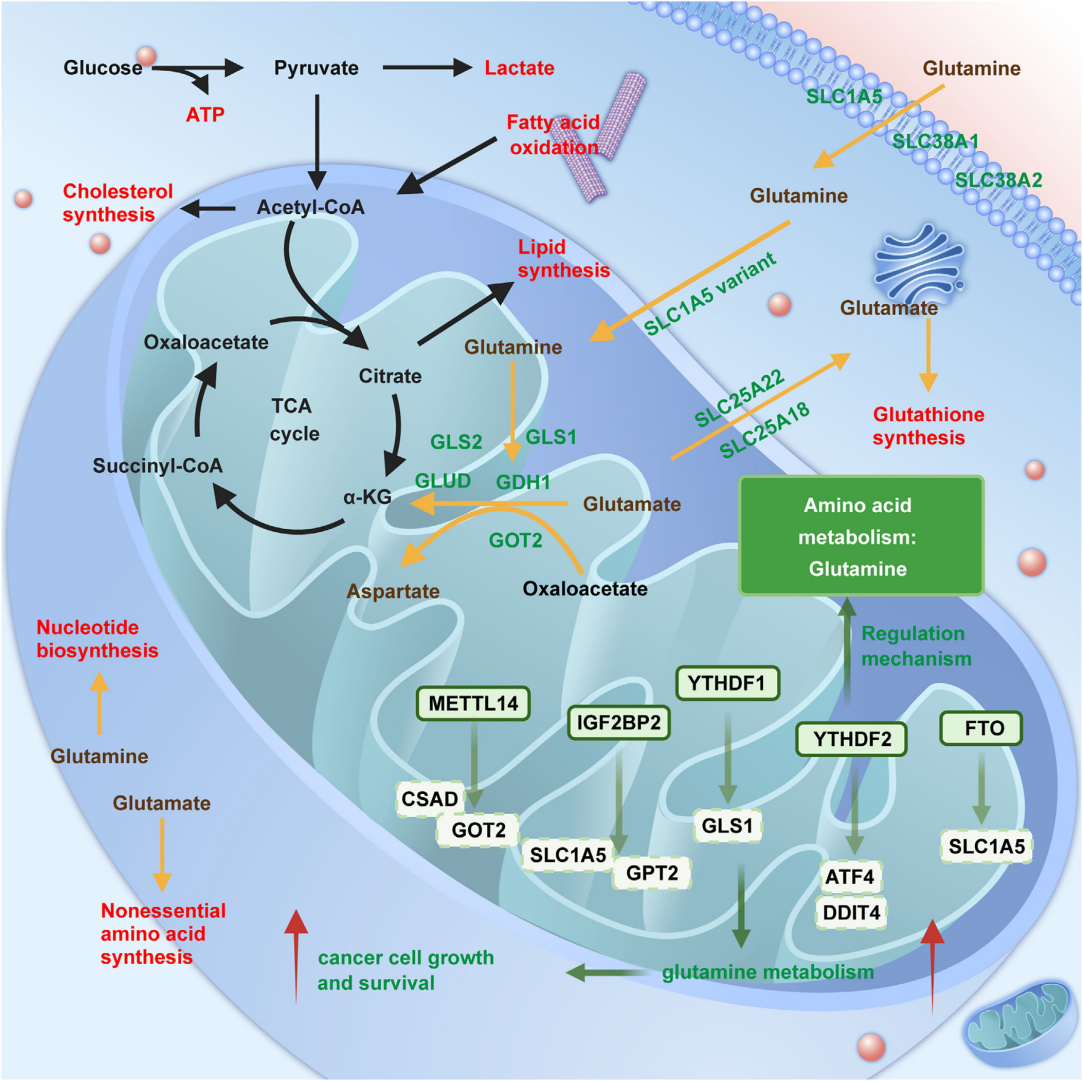
RNA methylation regulates the reprogramming of amino acid metabolism in multiple cancers. As the most abundant amino acid in the circulation, glutamine undertakes a versatile role in cell metabolism, participating in the supplementation of tricarboxylic acid (TCA) cycle and the biosynthesis of nucleotides, glutathione, and other nonessential amino acids. Given the energy‐generating and biosynthetic roles of glutamine in growing cells, cancer cells manipulate oncogenic alterations of glutamine metabolism through the regulation of RNA methylation to address the increased requirements for growth and proliferation. In acute myeloid leukemia, IGF2BP2 enhances the expression of glutamic pyruvate transaminase 2 (GPT2), and solute carrier family 1 member 5 (SLC1A5), thus driving glutamine metabolism. In colorectal cancer, YTHDF1 promote glutaminase 1 (GLS1) activity and increase glutamine uptake, while YTHDF2 elevates activating transcription factor 4 (ATF4) and DNA damage‐inducible transcript 4 (DDIT4) during the glutaminolysis inhibition. In clear cell renal cell carcinoma, FTO targets the glutamine transporter SLC1A5 to increase the glutamine consumption. Moreover, METTL14 participates in the regulation of glutamic‐oxaloacetic transaminase 2 (GOT2), and cysteine sulfinic acid decarboxylase (CSAD) during the progression of hepatocellular carcinoma.

Besides glucose metabolism, RNA methylation also possesses vital roles in Gln metabolism of multiple cancers. Recent studies showed that IGF2BP2 enhances the expression of MYC, glutamic pyruvate transaminase 2 (GPT2), and solute carrier family 1 member 5 (SLC1A5), thus promoting Gln metabolism in AML.^398^ Elevated levels of YTHDF1 in CRC have been validated to promote GLS1 activity, leading to increased Gln uptake and cisplatin chemoresistance. The role of YTHDF1 in mediating cisplatin resistance through enhanced Gln consumption has been further validated in a xenograft mouse model. The in vivo experimental data support that YTHDF1 silencing combined with cisplatin treatment demonstrates a reduction in cisplatin resistance by attenuating YTHDF1‐mediated Gln metabolism.^399^ Furthermore, the upregulation of activating transcription factor 4 (ATF4) by YTHDF2 results in an increase in the transcript levels of DNA damage‐inducible transcript 4 (DDIT4), an mTOR inhibitor, thereby facilitating prosurvival autophagy during glutaminolysis inhibition in CRC.^400^ In clear cell RCC (ccRCC) with VHL deletion or mutation, upregulated FTO targets the Gln transporter SLC1A5 to increase the Gln consumption rate and the sustain growth and survival of ccRCC cells.^401^ However, it should be noted that METTL14 participates in the regulation of mRNA involved in glutamate metabolism, including glutamic‐oxaloacetic transaminase 2 (GOT2), cysteine sulfinic acid decarboxylase (CSAD), and SOCS2, to suppress HCC progression (Figure 4).^402^ Overall, these findings showcase the crucial roles of RNA methylation in orchestrating Gln metabolism in cancer cells through various mechanisms. The intricate interplay between RNA methylation and aberrant Gln metabolism in cancer cells holds therapeutic potential across different types of cancers.

#### 4.2.3 Lipid metabolism and RNA methylation in cancer

**FIGURE 5:**
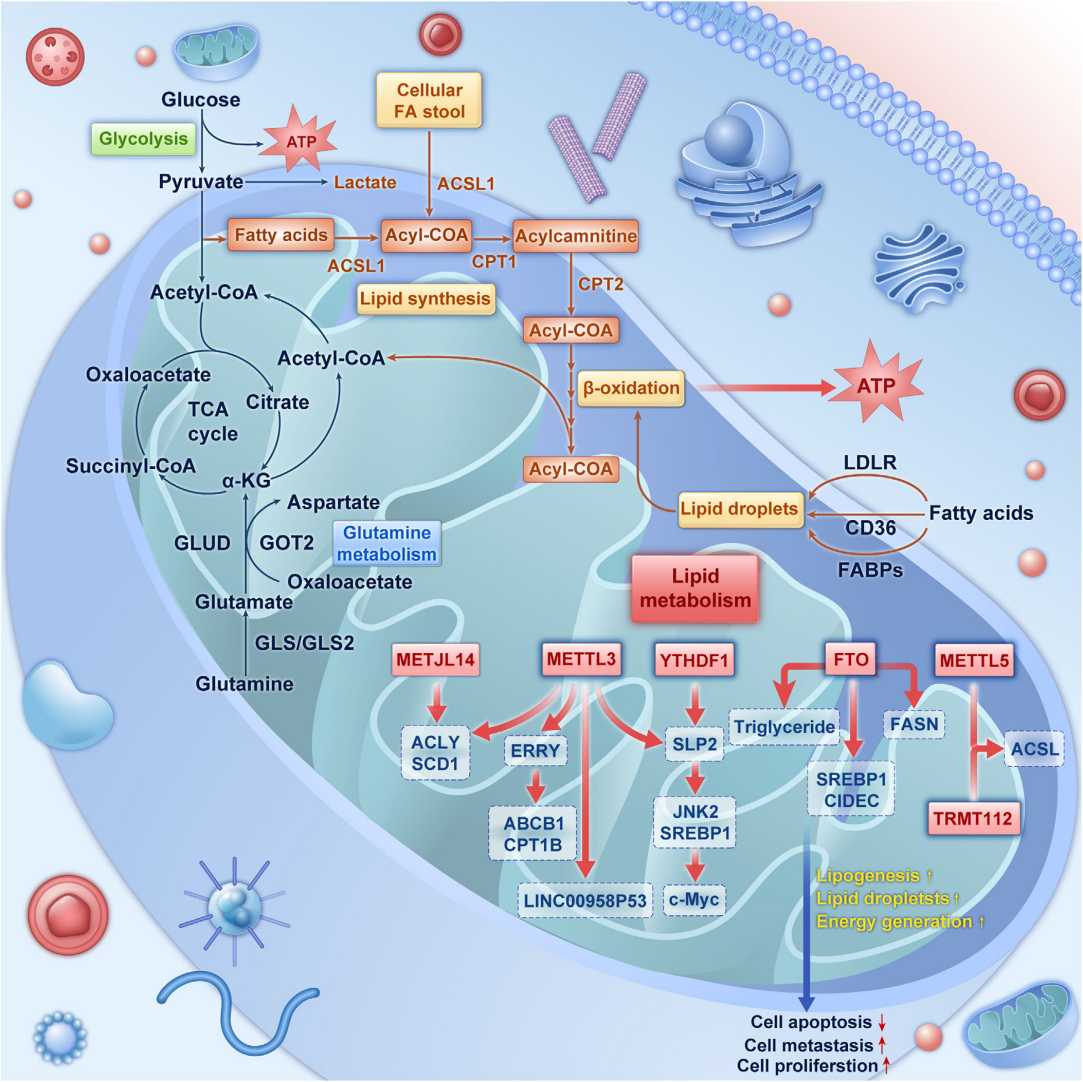
In liver cancer, RNA methylation is involved in the regulation of lipid metabolism to boost cancer development. The liver is the principal metabolizing organ. Liver cancer cells exhibit increased uptake of lipids, de novo fatty acid synthesis, lipid storage, and even fatty acid oxidation to meet the high demands of substrate and energy for cell proliferation and to adapt to a nutrient‐deficient microenvironment. In liver cancer, RNA modification plays a critical role in the modulation of lipid metabolism and triggers cancer progression. The m^6^A reader YTHDF1 and m^6^A writers (including METTL3, METTL5, and METTL14), upregulate the expression of RNA associated with lipid metabolism and result in enhanced lipogenesis. The m^6^A eraser FTO also upregulates lipid metabolism and contributes to cancer progression by modifying targets associated with the process of lipogenesis. FA, fatty acid; ACSL, acyl‐CoA synthetase long chain family member, CPT1, carnitine palmitoyltransferase 1; LDLR, low density lipoprotein receptor; FASPs, fatty acid synthesis proteins; ACLY, ATP citrate lyase; SCD1, stearoyl‐CoA desaturase 1; ERRγ, estrogen receptor‐related receptor γ; ABCB1, ATP binding cassette subfamily B member 1; CPT1B, carnitine palmitoyltransferase 1B; SLP2, stomatin‐like protein 2; JNK2, c‐Jun N‐terminal kinase 2; SREBP1, sterol regulatory element binding protein‐1; FASN, FA synthase; CIDEC, cell death‐inducing DFFA‐like effector C.

The liver is the principal lipid‐metabolizing organ in the body. In HCC, owing to increased modification of METTL3/14, the expression of ATP citrate lyase (ACLY) and stearoyl‐CoA desaturase 1 (SCD1) is upregulated, which contributes to increased lipid production and the accumulation of lipid droplets (Figure 5).^403^ The upregulation of METTL5 and TRMT112 expression in HCC samples is positively correlated with poor prognosis and mediates 18S rRNA m^6^A modification, thereby facilitating de novo lipogenesis via the overexpression of the acyl‐CoA synthetase long‐chain family (ACSL) in HCC cells.^404^ It has also validated that stomatin‐like protein 2 (SLP2), which is upregulated by METTL3 and YTHDF1, binds to the C‐terminal of c‐Jun N‐terminal Kinase 2 (JNK2) to increase JNK2 levels and subsequently enhance SREBP1 activity, thereby facilitating de novo lipogenesis in HCC. The SLP2 overexpression HCC animal model further confirmed the enhanced METTL3‐induced lipid metabolism and its carcinogenic functions.^405^ Functional experiments have revealed that METTL3 upregulates INC00958, which then interacts with miR‐3619‐5p to elevate the levels of HDGF, thereby promoting lipogenesis and cancer progression in HCC.^406^ FTO has been reported to increase the levels of FA synthase (FASN), SREBP1c, and cell death‐inducing DFFA (DNA fragmentation factor‐α)‐like effector c, decrease mitochondrial content, and promote triglyceride deposition, thereby contributing to the formation of lipid droplets and lipid accumulation.^407^, ^408^, ^409^ Additionally, METTL3 overexpression is associated with increased estrogen receptor‐related receptor γ (ERRγ) levels in chemo‐resistant HCC and BC cells. ERRγ binds to p65 to upregulate ATP binding cassette subfamily B member 1 (ABCB1) and the rate‐limiting FAO enzyme carnitine palmitoyltransferase 1B (CPT1B), thereby enhancing FAO and drug resistance in cancer cells.^410^ In CRC, the overexpression of DEGS2, a key ceramide‐synthesizing enzyme, results in the inhibition of ceramide synthesis and the proliferation and migration of CRC cells through reduced m^6^A modification of METTL3 and YTHDF2.^411^ In EC, FTO, along with YTHDF1 upregulates the expression of 17beta‐hydroxysteroid dehydrogenase 11 (HSD17B11), thereby promoting the formation of lipid droplets in EC cells and cancer development, which is associated with a poor prognosis for patients with EC.^412^ Moreover, increased HNRNPA2B1 levels upregulate the expression of the FA synthetic enzymes ACLY and acetyl‐CoA carboxylase 1 (ACC1) and subsequently contribute to lipid accumulation in EC.^413^ In AML, IGF2BP2 stabilizes protein arginine methyltransferase 6 (PRMT6) and subsequently downregulates the expression of the lipid transporter major facilitator superfamily domain containing 2A (MFSD2A), thereby maintaining the function of leukemia stem cells.^414^ In CC, depleted levels of the demethylase ALKBH5 indicate an unfavorable prognosis, and ALKBH5 functionally suppresses the expression of silent mating type information regulation 2 homologue 3 and ACC1 in an IGF2BP1‐dependent manner, ultimately inhibiting FA synthesis.^415^ In bladder cancer, METTL14 induces lncDBET overexpression, and along with FA binding protein 5 (FABP5), it promotes lipid metabolism and the malignant progression of bladder cancer.^416^ In GBM, constitutively activated EGFR/SRC/ERK pathway results in YTHDF2 overexpression and subsequently downregulate LXRA and HIVEP2 expression, thereby disrupting cholesterol homeostasis and sustaining GBM tumorigenesis.^417^

The m^5^C modification was recently found to regulate diverse biological processes involved in lipid metabolism. Studies have revealed that the m^5^C methyltransferase NSUN2 inhibits adipogenesis by accelerating the cell cycle of preadipocytes during the early stage of differentiation.^418^ The m^5^C reader ALYREF has also been found to repress adipogenesis and lipid accumulation by downregulating the expression of adipogenic differentiation marker genes.^419^ These findings are expected to shed light on the role of the m^5^C modification in cancer lipid metabolic reprogramming. In recent years, a study concerning osteosarcoma has shown that upregulated NSUN2 positively mediates lipid metabolism and cell proliferation of osteosarcoma cells through the overexpression of FABP5.^420^ Moreover, the m^1^A methyltransferase complexes TRMT6 and TRMT61A have been demonstrated to increase the m^1^A methylation of tRNA, thereby triggering PPARδ and Hedgehog signaling, which in turn promote cholesterol synthesis and self‐renewal of liver cancer stem cells.^421^ In general, these discoveries underscore the essential functions of RNA methylation in coordinating lipid metabolism to fulfill the increased energy demands of cancer cells through a variety of mechanisms. The regulatory mechanisms of RNA methylation on altered lipid metabolism in cancer cells presents a promising avenue for advancing cancer treatment.

#### 4.2.4 Other metabolic processes and RNA methylation in cancer

In addition to the regulation of the metabolism of three major nutrients during carcinogenesis, emerging evidence demonstrates that RNA methylation actively modulates other types of metabolism, such as mitochondrial metabolism and iron metabolism. Regarding mitochondrial metabolism, the RNA‐binding protein RALY upregulates a set of miRNAs, including miR‐676, miR‐483, and miR‐877, as a result of modification by METTL3, and downregulates the expression of electron transport chain genes, such as ATP5G3, ATP5I, ATP5G1, and CYC1, which are involved in the ROS‐related stress signal of mitochondrial metabolism in CRC.^422^ In GC, FTO increases the rate of degradation of the complete membrane protein caveolin‐1, thereby modulating mitochondrial fission/fusion and metabolism.^423^ METTL3 has been suggested to cooperate with IGF2BP1 to upregulate NDUFA4 expression and promote mitochondrial fission in GC.^371^ In RCC, the mitochondrial enzyme MTHFD2 modulates m^6^A methylation of HIF‐2α mRNA, thereby contributing to the metabolic reprograming of RCC.^424^ FTO downregulation is also associated with poor survival of patients with ccRCC and upregulates the expression of a central regulator of mitochondrial function, namely PPARδ coactivators‐1α (PGC‐1α), to induce oxidative stress and suppress ccRCC growth.^425^ In BC, METTL3 increases adenylate kinase 4 (AK4) expression to elevate ROS and p38 activity and augment tamoxifen resistance in cancer cells.^426^ Recently, a study reported that the m^1^A eraser ALKBH7 reduces the steady‐state levels of mt‐RNAs to enhance mitochondrial activity and upregulate OXPHOS.^151^ Additionally, recent evidence suggests a close association between m^6^A modification and iron metabolism during tumorigenesis.^427^ In hypopharyngeal squamous cell carcinoma, YTHDF1, upregulates TFRC expression to increase iron uptake and is positively correlated with ferritin levels and intracellular iron concentrations.^427^ The m^6^A demethylase ALKBH5 has been reported as a favorable prognostic marker for PDAC and to upregulate FBXL5 to degrade iron regulatory protein 2 and reduce intracellular iron levels.^428^

### 4.3 Clinical significance of metabolism‐focused RNA methylation in cancer

Research has provided strong evidence for the vital influence of RNA methylation on cancer cell metabolism, influencing processes such as glycolysis, glutaminolysis, lipid metabolism, and other key metabolic pathways. Dysregulation of RNA methylation in cancer cells impacts critical metabolic pathways, contributing to tumor progression, therapy resistance, and patient prognosis.^428^ Growing evidence reveals the aberrant expression of RNA modification and its modified proteins in diverse cancers and these abnormal RNA modification levels are evaluated to be associated with the prognosis of cancer patients.^429^, ^430^, ^431^, ^432^, ^433^ The different expression patterns of RNA modification regulators between cancers and para‐cancerous tissues enable researchers to accurately make a differential diagnosis of benign and malignant diseases.^434^, ^435^ The relationship between RNA modification levels and patient prognosis presents great opportunities for patients with more precise prognosis evaluation and personalized therapies.^436^, ^437^, ^438^, ^439^ Additionally, in the era of precision medicine, the clinical significance of metabolism‐focused RNA methylation extends to the potential development of personalized treatment strategies. Although current research is still in early stages and primarily focuses on basic investigations, numerous in vitro and in vivo assays have shown exhibited results for therapeutic approaches targeting RNA methylation in overcoming drug resistance in several cancer treatments.^440^ Exploring the mechanism by which RNA methylation affects tumor metabolism is beneficial for the development of targeted RNA methylation for tumor treatment. RNA methylation is regarded as important carcinogenic factors of clinical and functional significance. Several key molecules involved in RNA modification pathways have been identified as potential therapeutic targets in diverse cancers, including CRC, HCC, GC, BC, AML, CC, and PC. This section summarizes the progress made in research aimed at targeting RNA methylation to disrupt metabolic reprogramming and achieve the regulation of cancer progression. Additionally, we provide an overview of the performance of RNA methylation as a prognosis predictor and treatment target in several cancer types (Table 2).

**TABLE 2:** Clinical implications of RNA methylation in cancer metabolic reprogramming.

Cancer type	RNA‐modified regulators	Mechanism	Type of enzyme	Cells/organisms	Effect of gene knockdown	Inhibitor	Clinical implications	References
CRC	METTL3	METTL3/GLUT1/mTORC1 signaling pathway	Writer	CRC cell lines (HCT116, SW480, DLD1, POP66, and POP92), patient‐derived CRC organoids, Mettl3‐knockout mouse models, and human CRC tissues	Mettl3 knockout inhibited colorectal tumorigenesis.	/	Poor prognostic biomarker and therapeutic target	^349^
CRC	METTL3, YTHDF1	METTL3/YTHDF1/JAK1‐STAT3 signaling	Writer/Reader	Cell lines (HEK293T, Lewis lung carcinoma, murine bone‐marrow‐derived macrophages, B16F10, and MC38 cells), mouse models, and human CRC tissues	Mettl3 deficiency in myeloid cells alleviated the immunosuppression of TME.	/	Poor prognostic biomarker and immunotherapeutic strategy	^353^
CRC	METTL3	METTL3/LDHA	Writer	CRC cell lines (HCT‐116, SW480, and SW620) and mouse models	Targeted inhibition of METTL3 increased 5‐FU sensitivity of CRC cells.	/	Therapeutic target	^350^
CRC	WTAP	WTAP/NT5DC3/HKDC1	Writer	CRC cell lines (GES1, MGC803, HK2, SW839, NCM460, HT29, HCT116, and SW620), T2D mouse with xenografted colon tumor models, and clinical blood samples	WTAP‐regulated NT5DC3 level might be used to distinguish whether T2D patients are susceptible to developing CRC.	/	Predictive factor	^359^
HCC	METTL3	METTL3/mTOR signaling pathway	Writer	HCC cell lines (Huh‐7 and SMMC‐7721)	Downregulation of METTL3 inhibited tumor growth in vitro.	/	Poor prognostic biomarker and therapeutic target	^360^
HCC	ALKBH5	ALKBH5/UBR7/Keap1/Nrf2/Bach1/HK2	Eraser	HCC cell lines (Huh‐7, MHCC‐97L, HepG2, BEL‐7402, and BEL‐7404), and mouse models	ALKBH5/UBR7/Keap1/Nrf2/Bach1/HK2 axis provided a potential therapeutic target for the HCC treatment.	/	Poor prognostic biomarker and therapeutic target	^368^
HCC	METTL5, TRMT112	METTL5/TRMT112/ACSL	Writer	HCC cell lines (HepG2, Huh7, Hep3B, MHCC97H, SNU‐449, and PLC/PRF/5), mouse models, and human HCC tissues	METTL5 knockdown blocked HCC tumorigenesis in vitro.	/	Poor prognostic biomarker and therapeutic target	^404^
GC	METTL3, IGF2BP3	METTL3/IGF2BP3/HDGF/GLUT4/ENO2	Writer/Reader	GC cell lines (BGC823, AGS, HGC‐27, and NCI‐N87), xenograft models, organoids, and human GC tissues	METTL3 knockdown suppressed cell viability, angiogenesis, tumor growth, and liver metastasis.	/	Poor prognosis and therapeutic target	^370^
GC	WTAP	WTAP/HK2	Writer	GC cell lines (BGC‐823, MGC‐803, AGS, SGC‐7901), xenograft mouse models, and human GC tissues	WTAP knockdown suppressed the tumor growth in vivo.	/	Poor prognostic biomarker and therapeutic target	^376^
GC	IGF2BP3	IGF2BP3/c‐MYC/LOC101929709/LIN28B/PI3K–AKT pathway	Reader	GC cell lines (AGS, MKN1, SGC‐7901, BGC‐823, and MGC‐803), and mouse models	repressing IGF2BP3/c‐MYC/LOC101929709/LIN28B/PI3K–AKT pathway inhibited the Warburg effect and tumor progression.	/	Therapeutic target	^372^
EC	METTL3, YTHDF	METTL3/YTHDF/APC/β‐catenin/cyclin D1/c‐Myc/PKM2	Writer/Reader	EC cell lines (TE1, TE10, KYSE30, KYSE70, KYSE140, KYSE150, KYSE180, KYSE410, KYSE450, and Het‐1a), mouse models, and human EC tissues	METTL3 depletion increased APC expression and reduced tumor size of athymic nude mice.	/	Poor prognostic biomarker	^392^
EC	FTO, YTHDF1	FTO/YTHDF1/HSD17B11	Eraser/Reader	EC cell lines (KYSE510 and TE1), mouse models, and human EC tissues	FTO knockdown inhibited the proliferation, invasion, stemness, and tumorigenicity of EC cells.	/	Poor prognostic biomarker and therapeutic target	^412^
EC	ALKBH5, HNRNPA2B1	ALKBH5/HNRNPA2B1/ACLY/ACC1	Eraser/Reader	EC cell lines (ECA109 and TE10) and human EC tissues	Knockdown of HNRNPA2B1 inhibited the expression of de novo fatty acid synthetic enzymes and cellular lipid accumulation.	/	Poor prognostic biomarker and therapeutic target	^413^
RCC	METTL14	METTL14/BPTF	Writer	RCC cell lines (786‐O, ACHN, and Caki‐1), xenograft models, organoids, and human RCC tissues	METTL14 deficiency promoted RCC metastasis.	Bromodomain PHD finger transcription factor inhibitor AU1	Favorable prognostic biomarker and therapeutic target	^387^
RCC	FTO	FTO/PGC‐1α	Eraser	RCC cell lines (786‐O, and 769‐P), xenograft models, and human RCC tissues	FTO knockdown resulted in increased cell proliferation and reduced cell apoptosis.	Meclofenamic acid	Favorable prognostic biomarker and therapeutic target	^425^
Cervical cancer	METTL3, YTHDF1	METTL3/YTHDF1/HK2	Writer/Reader	CC cell lines (CaSki, SiHa, C33A, and HT‐3), xenograft models, and human RCC tissues	METTL3 knockdown inhibited cell proliferation, glycolysis, and tumor growth.	/	Poor prognostic biomarker	^384^
Cervical cancer	IGF2BP2	HPV E6/E7/IGF2BP3/c‐MYC/LOC101929709/LIN28B/PI3K–AKT pathway	Reader	CC cell lines (SiHa and HeLa) and xenograft models	IGF2BP2 knockdown attenuated the aerobic glycolytic capacity and growth of CC cells.	/	Poor prognostic biomarker and therapeutic target	^385^
Cervical cancer	ALKBH3 and YTHDF1	ATP5D and E2F1	Eraser/Reader	Cervical cancer cell lines (5637 and T24) and human cervical cancer tissues		/	Poor prognostic biomarker	^397^
PDAC	IGF2BP2	IGF2BP2/GLUT1	Reader	PDAC cell lines (BxPC‐3, Capan‐1, MIA PaCa‐2, PANC‐1, and SW1990), xenograft models, and human PDAC tissues	IGF2BP2 knockdown suppressed cell proliferation and aerobic glycolysis.	/	Poor prognostic biomarker and therapeutic target	^379^
Lung cancer	FTO, YTHDF1	FTO/YTHDF1/c‐Myc/WNT/β‐catenin	Eraser/Reader	Lung cancer cell lines (H322 and HEK 293T), xenograft models, and human lung adenocarcinoma tissues	FTO downregulation promoted tumor cell glycolysis, growth, metastasis, and tumorigenesis in mice.	/	Favorable prognosis	^393^
Glioblastoma	YTHDF2	YTHDF2/LXRα/HIVEP2/EGFR/SRC/ERK signaling	Reader	Cell lines (glioma Hs683 and SW1783 cell lines and GBM T98G, U87 MG, LN229) and xenograft models	YTHDF2 loss in the GSC cells inhibited proliferation, invasion, and tumorigenicity of GBM cells.	/	Poor prognostic biomarker	^417^
AML	IGF2BP2	IGF2BP2/MYC/GPT2/SLC1A5	Reader	AML cell lines (U937, Kasumi‐1, and HEK293T), xenograft models, and human AML cell samples	IGF2BP2 knockdown inhibited the maintenance and progression of AML.	CWI1‐2	Poor prognostic biomarker and therapeutic target	^398^
Bladder cancer	ALYREF	HIF‐1α	Reader	Bladder cancer cell lines (5637 and T24) and human bladder cancer tissues	ALYREF knockdown inhibited glycolysis metabolism, cell proliferation, tumor volume, and weight of bladder cancer.	/	Poor prognostic biomarker	^395^
Osteosarcoma	NSUN2	FABP5	Writer	Osteosarcoma cell lines (U937, Kasumi‐1, and HEK293T), xenograft models, human osteosarcoma tissue samples	NSUN2 deficiency inhibited fatty acid metabolism and the proliferation, migration, and invasion of osteosarcoma.	Etomoxir	Poor prognostic biomarker and therapeutic target	^420^

CRC, colorectal cancer; HCC, hepatocellular carcinoma; GC, Gastric cancer; PDAC, pancreatic ductal adenocarcinoma; RCC, renal cell carcinoma; AML, acute myeloid leukemia; ESCC, esophageal squamous cell carcinoma; EC, endometrial cancer; GLUT1, glutamate dehydrogenase 1; TME, tumor microenvironment; LDHA, lactate dehydrogenase A; NT5DC3, 5′‐nucleotidase domain‐containing protein 3; HKdc1, hexokinase domain component 1; T2D, type 2 diabetes; UBR7, ubiquitin protein ligase E3 component N‐recognin 7; Keap1, kelch‐like ECH‐associated protein 1; Nrf2, nuclear factor erythroid 2‐related factor 2; Bach1, BTB domain and CNC homolog 1, HK2, hexokinase 2; ACSL, acyl‐CoA synthetase long‐chain family; HDGF, heparin binding growth factor; ENO2, enolase 2; APC, adenomatous polyposis coli; PKM2, pyruvate kinases type M2; HSD17B11, 17beta‐hydroxysteroid dehydrogenase 11; ACLY, ATP citrate lyase; ACC1, acetyl‐CoA carboxylase 1; BPTF, bromodomain PHD finger transcription factor; PGC‐1α, PPARδ coactivators‐1α; ATP5D, the δ subunit of ATP synthase; E2F1, E2F transcription factor 1; LXRα, liver X receptor alpha; HIVEP2, human immunodeficiency virus type I enhancer binding protein 2; GPT2, glutamic pyruvate transaminase 2; SLC1A5, solute carrier family 1 member 5; FABP5, FA binding protein 5.

#### 4.3.1 Clinical significance of m6A modification in CRC

One such molecule is METTL3, increased METTL3 levels indicate poor prognosis in patients with CRC, including shorter disease‐free intervals, and undesirable clinicopathological factors such as pathological differentiation, AJCC stage, and disease recurrence.^349^, ^353^, ^441^ Currently, 5‐FU is the most prevalent chemotherapeutic agent employed in CRC therapy. Several in vitro and in vivo experiments have indicated that 5‐FU‐resistant CRC cells exhibit METTL3 upregulation and aberrant activation of glycolysis. Inhibiting the METTL3/LDHA axis has been validated to effectively resensitize 5‐FU‐resistant CRC cells to 5‐FU. This finding presents a potential strategy to restore 5‐FU efficacy in CRC.^350^ Targeting METTL3 also decreases glucose uptake, impairs cell proliferation, and inhibits cancer growth, providing novel perspectives for CRC therapeutic strategies.^352^ Another molecule of interest is METTL16, which is highly expressed in CRC tissues and cell lines. Elevated METTL16 expression is positively related to deleterious clinicopathological parameters, such as tumor size, lymph node metastasis, and distant metastasis of patients with CRC. Further analysis applying multivariate Cox regression also validates the independent performance of METTLE16 for prognosis prediction in CRC.^442^ Another study conducted at the Sun Yat‐sen University Cancer Center has reported a negative correlation between the expression of LINRIS and IGF2BP2 and prognosis of patients with CRC. Two patient‐derived xenograft models of type 2 diabetes (T2D) with xenografted colon tumors under high glucose concentrations, including the C57BL/6 and the BALB/c nude mouse model, have provided further evidence that inhibiting the LINRIS/IGF2BP2/MYC axis effectively suppresses cancer growth without notable side effects, indicating a potential treatment approach for CRC.^355^ Lactoferrin suppresses the m^6^A modification of NT5DC3 mediated by WTAP, leading to the inhibition of CRC progression in both two types of mouse models. Moreover, analysis of clinical blood samples also suggests that NT5DC3 levels could be used as a predictive marker for CRC occurrence in patients with T2D.^359^ In addition, the overexpression of YTHDF1 notably enhances cisplatin resistance in cisplatin‐resistant CRC cells (LoVo CDDP R) and in vivo xenograft mouse models by upregulating GLS1‐induced Gln metabolism. Several experiments have validated that the inhibition of YTHDF1 and cisplatin synergistically perform tumor‐suppressive functions.^399^ These findings underscore the importance of RNA modifications, such as m^6^A methylation, in CRC development and progression. Targeted therapies against specific molecules involved in RNA modification pathways hold promise for the development of innovative treatment approaches for CRC.

#### 4.3.2 Clinical significance of m6A modification in HCC

Moreover, METTL3 has been found to upregulate LINC00958 and promote lipogenesis in HCC. In HCC mouse models, a nanoplatform based on si‐LINC00958 for HCC systemic delivery has exhibited superior efficacy and safety as a systemic delivery option for HCC treatment. This approach shows promise in effectively targeting the aberrant lipogenesis pathway to inhibit tumor growth and progression in HCC.^406^ Likewise, YTHDF1 upregulates SLP2 expression in a METTL3/m^6^A‐dependent manner, which is correlated with the overall and disease‐free survival in patients with HCC. Overexpression of SLP2 enhances lipid synthesis in HCC cells by regulating JNK2, thereby promoting cancer proliferation and metastasis. The aforementioned findings highlight the potential of targeting the expression pattern of SLP2 as a viable strategy for HCC treatment.^405^ Moreover, an established m^1^A‐related scoring system based on the metabolic characteristics of HCC was validated as a reliable tool for evaluating drug sensitivity of patients and facilitating risk stratification.^396^ These recent discoveries not only shed light on the role of RNA modifications and their impact on HCC development and treatment but also present potential avenues for targeted therapies and precision medicine approaches in managing this challenging disease.

#### 4.3.3 Clinical significance of RNA methylation in GC

It has also been revealed that METTL14 interferes with the aerobic glycolysis process in GC cells by mediating the m^6^A modification of phospholysine phosphohistidine inorganic pyrophosphate Previous studies have identified LHPP as a histidine phosphatase and confirmed its role as a tumor suppressor in various cancer types.^443^, ^444^, ^445^ Recent research has revealed that METTL14 regulates LHPP through m^6^A modification, leading to the inhibition of GSK‐3β acetylation. This, in turn, suppresses the HIF‐1α‐induced aerobic glycolysis, as well as the subsequent proliferation and invasion of GC HGC‐27 cells.^377^ The observed tumor suppression effects demonstrated by METTL14 and LHPP highlight the potential of m^6^A methylation as a viable therapeutic approach for GC treatment. Additionally, increased expression of IGF2BP1 in GC tissues has been associated with enhanced aerobic glycolysis, aggressive behaviors of GC cells, and poor prognosis in GC patients. In vivo experiments have shown that knockdown of IGF2BP1 suppresses GC carcinogenesis, offering a promising target for GC treatment. These findings provide important insights into potential therapeutic interventions for GC.^373^ Furthermore, it has been found that METTL3 induces the upregulation of RPRD1B, which enhances FA uptake and synthesis, as well as lymph node metastasis of GC, through the c‐Jun/c‐Fos/SREBP1 axis. The disruption of this functional circuitry is observed to achieve the impairment of FA metabolism and lymph node metastasis.^446^ The association between METTL3 and FA metabolism presents a new therapeutic target for GC. Moreover, glutathione, a natural antioxidant known for its critical role in cellular antioxidative systems, has been implicated in excessive ROS clearance and maintenance of redox status.^447^, ^448^, ^449^, ^450^, ^451^ Given its crucial functions of cellular antioxidative system, glutathione‐depleting strategy has garnered much attention in cancer therapy.^452^, ^453^, ^454^, ^455^ Additionally, the curcumin analog WZ35 induces multiple metabolic remodeling processes that suppress GC cell metastasis and growth. Mechanistically, WZ35 increases the m^6^A levels in GLS2 levels to deplete intracellular glutathione through upregulating YAP, AXL, and ALKBH5 expression.^456^ The involvement of ALKBH5 expression in WZ35‐induced glutathione depletion uncovers new potential targets for GC treatment. Moreover, integrative proteomics analysis of METTL3 overexpressing GC BGC‐823 cells has revealed an association between METTL3 and proteins involved in mitochondria‐related OXPHOS. Notably, METTL3 upregulates AVEN and DNAJB1 levels, contributing to a poorer clinicopathological grade and stage in GC patients. These findings suggest that METTL3 influences GC cell metabolism, particularly OXPHOS, and hold promise for developing novel therapeutic targets and predictive markers in GC.^457^ Additionally, the upregulation of IGF2BP1 in GC tissues is associated with enhanced aerobic glycolysis and malignant behaviors of GC cells and with a poor prognosis in patients with GC. In vivo experiments have shown that IGF2BP1 knockdown suppresses the carcinogenesis of GC. This finding provides critical insights into therapeutic interventions for GC.^373^ These findings offer valuable insights into the interplay between RNA methylation and cancer metabolism, providing potential avenues for therapeutic interventions and prognostic predictions in GC.

#### 4.3.4 Clinical significance of m6A modification in CC

Moreover, in CC, upregulated IGF2BP2 expression in CC tissues is positively associated with the tumor stage. HPV 16/18 E6/E7 activates IGF2BP2 and regulates MYC methylation to facilitate aerobic glycolysis and cancer progression in CC. The correlation between HPV E6/E7 and aerobic glycolysis may serve as a potential avenue for CC treatment.^385^ Furthermore, the Kaplan–Meier survival curves based on TCGA datasets indicate that patients with CC who exhibit ALKBH3 upregulation show poorer overall survival, suggesting its implication in prognosis prediction.^397^ Thus, strategies such as genetic knockout, pharmacological inhibition, or interference with these molecules significantly reverse drug resistance and improve the treatment outcomes for refractory BC. Moreover, understanding the role of HPV E6/E7 and IGF2BP2 in regulating aerobic glycolysis opens up new possibilities for targeted therapies in CC. The upregulation of ALKBH3 in CC also indicates its potential as a prognostic biomarker that could aid in predicting overall survival.

Moreover, CWI1‐2, a small‐molecule compound, was recently identified to suppress IGF2BP2 and its functional targets (MYC, GPT2, and SLC1A5) and disrupt the Gln pathway, thereby attenuating the colony‐forming and self‐renewal abilities of AML cells, delaying the onset of leukemia, and prolonging the survival of mice with MA9‐induced leukemia. Given the high significance of AML to Gln metabolism, CWI1‐2, which inhibits IGF2BP2, is an effective compound for AML treatment.^398^ Mutant IDH1/2 enzymes were previously reported to upregulate R‐2HG production, accompanying by the suppression of the FTO/m^6^A/MYC/CEBPA signaling axis. These changes cause the antileukemic effects including the inhibition of fat mass and leukemia cell viability, which provides a novel theoretical direction for leukemic therapy.^458^ Notably, patients with BC who exhibit ALKBH5 or GLUT4 overexpression typically show a poor prognosis and resistance to HER2‐targeted therapy. The significant reversal of resistance in BC cells to trastuzumab and lapatinib has been achieved through genetic knockout and pharmacological inhibition of GLUT4, as well as interfering with ALKBH5 to suppress GLUT4 expression. This effect provides a new breakthrough for improving the treatment of drug‐resistant BC.^383^ In addition, increased expression of ALKBH5 has been observed in PC tissues as well as in PANC‐1 and MIA PaCa‐2 cells exposed to hypoxia. Through the combined application of MeRIP‐seq and RNA‐seq technologies, it has been revealed that the m^6^A reader YTHDF2 enhances the stability of histone deacetylase type 4 (HDAC4), thus promoting glycolytic metabolism and migration of PC cells under hypoxic conditions. This forms a positive feedback loop comprising ALKBH5, HDAC4, and HIF‐1α, which mutually accelerates glycolytic metabolism to adapt to hypoxic conditions. Based on these findings, the blockade of the ALKBH5/HDAC4/HIF‐1α feedback loop represents a potentially novel approach for targeted treatment of hypoxia‐driven PC.^459^ These findings shed light on novel therapeutic approaches and prognostic markers by targeting m^6^A modification and m^6^A regulators for female reproductive system cancers, offering new avenues for improving treatment efficacy and patient outcomes.

#### 4.3.5 Clinical significance of other types of RNA modifications in cancer

The overexpression of the m^5^C reader ALYREF also exerts carcinogenic effects to promote glycolysis, which is implicated in the poor overall and recurrence‐free survival of patients with bladder cancer.^395^ Moreover, the increased expression of the m^5^C eraser NSUN2 is correlated with an unfavorable prognosis in patients with osteosarcoma. The FAO inhibitor etomoxir and FABP5 deficiency have proven effective in counterbalancing the impact of NSUN2 upregulation on FA metabolism and the proliferation, migration, and invasion of osteosarcoma 143b cells. Animal models have validated that the inhibition of the NSUN2/FABP5 axis attenuates the prooncogenic effects of m^5^C modification in osteosarcoma, thus presenting novel avenues for osteosarcoma treatment.^420^ Overall, these findings also underscore the importance of addressing m^5^C modifications in multiple types of cancer therapy. It is worth noting that the findings discussed in this section are based on current research and metabolism‐focused RNA methylation in cancer has substantial clinical significance, with the potential to impact cancer diagnosis, treatment, and patient outcomes. However, further investigations are necessary to fully elucidate the therapeutic potential of targeted RNA methylation and its application in disrupting cancer metabolic reprogramming.

## 5 ASSOCIATION OF RNA METHYLATION WITH CANCER IMMUNITY

In addition to cancer metabolism reprogramming, RNA methylations have been shown to influence tumor immune response in tumor progression. Growing evidence has shown the correlation between m^6^A methylation and immune landscape in diverse types of cancer.^460^ An increasing number of studies suggest that METTL3 plays a pivotal role in maintaining the homeostasis of immune cells.^460^, ^461^ It has been confirmed that METTL3 mediates the modification of specific mRNA transcripts within T cells, including SOCS family mRNAs, PD‐L1 mRNA, and mRNAs involved in interferon‐gamma and interferon‐beta pathways.^460^, ^462^, ^463^, ^464^, ^465^ For instance, METTL3 mediates the modification of circIGF2BP3 in combination with YTHDC1, and elevated circIGF2BP3 sponges miR‐328‐3p and miR‐3173‐5p to upregulate plakophilin 3 (PKP3) expression. Subsequently, PKP3 stabilizes only the deubiquitinating enzyme otubain‐1 (OTUB1) mRNA to inhibit the ubiquitination of PD‐L1, contributing to reduced CD8+ T cell infiltration in non‐small cell lung cancer.^466^ Multiple m^6^A methylation regulators have also been found to be highly expressed in ESCC tissues, along with high PD‐L1 expression. Moreover, copy number alterations of m^6^A regulators have been observed to significantly disrupt immune cell infiltration in the ESCC immune microenvironment, affecting B cells, CD4+T cells, CD8+T cells, neutrophils, and dendritic cells.^467^ Additionally, m^6^A has been implicated in the association between methionine metabolism and tumor immunity in tumor progression. YTHDF1 and methionine metabolism mediate the m^6^A modification of PD‐L1 and V‐domain Ig suppressor of T cell activation, therefore contributing to reduced CD8+T cell infiltration, immune surveillance escape, and antitumor immunity resistance.^468^ High WTAP expression also causes the suppressed infiltration of T lymphocytes in gastric cancer, especially T regulatory (Treg) and CD4 memory‐activated T cells.^469^ And m^6^A methylation has been found to involve in innate immunity evasion through the modification of circRNAs.^470^ Evidence has revealed that YTHDF2 attenuates the immunogenicity of circRNAs and induces the evasion of circRNAs away from innate immune response by acting with m^6^A modification.^471^ It has also demonstrated that loss of YTHDF1 in classical dendritic cells enhances the cross‐presentation of tumor antigens and CD8+T cell antitumor response through the recognition of methylated transcripts encoding lysosomal proteases.^51^ Furthermore, the deletion of ALKBH5 has been shown to hamper the accumulation of immunosuppressive Treg and myeloid‐derived suppressor cells in melanoma via the inhibition of monocarboxylate transporter 4 expression.^472^ And pharmacological inhibition of ALKBH5 has shown to prolong the survival of mice and enhance the effectiveness of anti‐PD‐1 immunotherapy.^473^

Similar to m^6^A modification, m^5^C RNA methylation is also required for the regulation of the immune microenvironment in diverse cancer types. Increasing evidence has identified multiple m^5^C RNA regulators are abnormally expressed in many cancer tissues and play important roles in modulating immune functions in cancer.^474^, ^475^, ^476^, ^477^ In triple‐negative BC, NSUN2 has been found to be expressed in monocytes/macrophages and NSUN6, on the other hand, is expressed in Treg cells.^478^ Additionally, NSUN6 expression has been shown to correlate strongly with multiple immune cells, particularly CD4+ T cells, suggesting a broader role in modulating immune responses.^479^ It has been also reported that NSUN2 triggers the translation of intercellular adhesion molecule 1 mRNA and further strengthens the adhesion of leukocytes to endothelial cells.^480^ Notably, NSUN2 expression level has been found to have an essential implication with T‐cell activation and patient survival in the development of HNSCC. Patients with high T‐cell activation status have worse overall survival when NSUN2 expression is low, suggesting that NSUN2 expression plays a crucial role in influencing T‐cell activation and subsequent antitumor immune responses in HNSCC.^481^ Emerging studies have also highlighted the influence of m^7^G methylation for the maturation and functions of immune cells and tumor immunity.^474^ The established m^7^G‐related risk score model has exhibited a strong association with immune cell infiltration, immune checkpoints and immune‐related signaling pathways in uterine corpus endometrial carcinoma.^482^ Moreover, recent research has also uncovered the negative association between m^1^A levels and CD8+ T effector cell proliferation in colon cancer.^195^ The m^1^A Score system based on the expression of the 71 m^1^A‐related genes offers an efficient insight for the identification of immune cell infiltration in TME and the development of antitumor immunotherapy strategies.

In summary, RNA modifications have been shown to affect various biological properties of immune cells, including differentiation, activation, and development, thus participating in the immune responses in diverse cancers. The intricate interaction between different patterns of RNA methylations and various immune cell types also profoundly influences the efficacy of antitumor immunotherapy targeting the PD‐L1 checkpoint. However, while the mechanism presented primarily focuses on m^6^A methylation in the tumor immune microenvironment, there is a need for more in‐depth understanding of the interaction network of other RNA methylation patterns on specific immune cells in cancer. Further research is essential for improved insight into this important aspect of cancer immunology.

## 6 CONCLUSION AND FUTURE PERSPECTIVES

RNA modification is a rapidly developing field within the context of epigenetic modifications in oncology. Accumulating evidence has validated that the aberrant expression of RNA modification regulators is closely associated with prognosis especially in cancer metabolism, suggesting that these regulators can serve as crucial indicators of prognosis in clinical settings.^483^ These alterations in cancer cell metabolism affects tumor growth, metastasis, and response to therapy. Therefore, understanding the clinical significance of metabolism‐focused RNA methylation is essential for developing targeted therapeutic strategies and improving patient outcomes. The current mechanistic findings of RNA methylation on cancer development provide a theoretical foundation and insights for treating diseases using inhibitors of RNA methylation. The role of RNA methylation in cancer metabolism and therapy resistance has been increasingly recognized, making it a potential target for precise cancer treatment.^441^, ^484^ Recent in vitro and in vivo assays have indicated encouraging outcomes for therapeutic strategies aimed at targeting RNA methylation.^472^, ^485^, ^486^ However, it is important to note that current research efforts pertaining to the role of RNA methylation in cancer metabolic reprogramming have primarily concentrated on basic research. Clinical trials of small‐molecule inhibitors of RNA methylation in cancer metabolic reprogramming are still in the early stages and have yet to reach the clinical application stage.^487^ In addition, the same writer, eraser, and reader proteins of RNA methylation display diverse biological functions and regulatory mechanisms that vary not only across different types of cancers but also within the same cancer type.^488^ This phenomenon is likely attributed to factors such as cancer heterogeneity, variations in sample size, differences in experimental approaches, and disparities in the cancer microenvironment. To advance our understanding, future studies should strive to unravel the precise mechanisms underlying the effects of RNA methylation on cancer metabolic reprogramming, taking into account these varying factors. Another important consideration is the cell or tissue specificity of RNA methylation. The development of cell‐ or tissue‐specific small‐molecule inhibitors targeting RNA methylation would be highly desirable. It is anticipated that preclinical findings related to targeted RNA methylation in cancer metabolism will eventually translate into clinical applications.^26^ Furthermore, the integration of artificial intelligence technology and chemosynthesis holds potential for the development of anticancer agents targeting RNA methylation in cancer metabolic reprogramming.^489^ This interdisciplinary approach could provide innovative solutions and contribute to the advancement of precision medicine in oncology. Overall, the rapidly evolving exploration of RNA modification in oncology has shown promising prospects for improving cancer prognosis and treatment. Through further research efforts, a deeper understanding of the underlying mechanisms and the development of targeted interventions can be achieved, leading to improved clinical outcomes in the future.

## AUTHOR CONTRIBUTIONS

W. Z. designed the study, and reviewed and edited the manuscript. G. L., Q. Y., and P. L. participated in original draft preparation. H. Z., Y. L., S. L., Z. L., and Y. S. collected the references and help with reviewing the manuscript. All authors have read and approved the article.

## CONFLICT OF INTEREST STATEMENT

There are no conflict of interest to declare.

## ETHICS STATEMENT

Not applicable.

## Data Availability

Not applicable.
